# Germline variants in *ATM*, *BRCA2*, other cancer predisposition and novel candidate genes are implicated in glioma risk in adult glioma patients with a familial or personal history of tumors

**DOI:** 10.1007/s00401-025-02972-6

**Published:** 2026-01-17

**Authors:** Frank Brand, Lily S. Rose, Amir H. Akbarzadeh, Christine A. M. Weber, Isabel Eckert, Gunnar Schmidt, Bernd Auber, Alisa Förster, Ulrike Beyer, Robert Geffers, Stephan Bartels, Michael Lalk, Manolis Polemikos, Michael Friese, Michael Sabel, Philipp Schwenkenbecher, Paul Kremer, Arya Nabavi, Amir Samii, Ulrich Lehmann, Guido Reifenberger, Joachim K. Krauss, Bettina Wiese, Christian Hartmann, Ruthild G. Weber

**Affiliations:** 1 Department of Human Genetics, Hannover Medical School, OE 6300, Carl-Neuberg-Str. 1, 30625 Hannover, Germany; 2 Department of Neurology, Henriettenstift, Diakovere Krankenhaus gGmbH, Hannover, Germany; 3 Department of Neurosurgery, Hannover Medical School, Hannover, Germany; 4 Genome Analytics Research Group, Helmholtz Centre for Infection Research, Braunschweig, Germany; 5 Institute of Pathology, Hannover Medical School, Hannover, Germany; 6 Department of Neurosurgery, KRH Klinikum Nordstadt, Hannover, Germany; 7 Department of Pathology and Neuropathology, Asklepios Klinik Nord—Heidberg, Hamburg, Germany; 8 Department of Neurosurgery, University Hospital Düsseldorf and Heinrich Heine University, Medical Faculty, Düsseldorf, Germany; 9 Beta Klinik, Bonn, Germany; 10 Department of Neurology, Hannover Medical School, Hannover, Germany; 11 Department of Neurosurgery, Asklepios Klinik Nord—Heidberg, Hamburg, Germany; 12 Department of Neurosurgery, International Neuroscience Institute, Hannover, Germany; 13 Institute of Neuropathology, University Hospital Düsseldorf and Heinrich Heine University, Medical Faculty, Düsseldorf, Germany; 14 German Cancer Consortium (DKTK), Partner Site Essen/Düsseldorf and German Cancer Research Center (DKFZ), Heidelberg, Germany; 15 Department of Neuropathology, Institute of Pathology, Hannover Medical School, Hannover, Germany

**Keywords:** Adult-type diffuse glioma, Cancer predisposition genes, Germline variants, *ATM*, *BRCA2*

## Abstract

Familial occurrence of gliomas has been reported in around 5% of patients. Studies on the genetic landscape of glioma predisposition are scarce. Here, leukocyte DNA of 213 adult glioma patients with a familial and/or personal tumor history from 206 families was subjected to whole-exome sequencing. Germline variants (GVs) were analyzed using two approaches: (1) GVs in 164 established cancer predisposition genes (CPGs) or suspected glioma risk genes were extracted and classified; (2) the enrichment of genes with loss-of-function or deleterious missense GVs that were ultrarare or ClinVar likely pathogenic/pathogenic in the glioma versus a control cohort (*n* = 391) was determined. In 23% (48/213) of glioma patients with a familial/personal tumor history, GVs predicted to be deleterious in CPGs were detected. Of the mutated CPGs, 37% were involved in DNA damage response, including *ATM*, *BRCA2*, *PMS2*, *POLE*. *ATM* GVs (*n* = 6) preferentially predisposed to IDH-mutant astrocytoma (*P* = 0.007) in patients that were significantly younger at diagnosis than patients without GVs (*P* = 0.022). *BRCA2* GVs (*n* = 5) were also significantly enriched in glioma patients in approach 2 (*P* = 0.005). The other mutated CPGs, glioma risk or enriched novel genes play roles in diverse processes, including metabolism and signal transduction. Syn-/metachronous non-brain tumors were diagnosed in 29% of glioma patients with GVs. In 11% of patients, the identified CPG GVs potentially sensitized to targeted therapies, such as PARP, immune checkpoint, or EGFR inhibitors. In conclusion, our study identifies CPGs and novel genes relevant in germline testing of glioma patients with a familial/personal tumor history, possibly resulting in targeted treatment options.

**Supplementary Information:**

The online version contains supplementary material available at 10.1007/s00401-025-02972-6.

## Introduction

Adult-type diffuse gliomas represent a group of primary brain tumors that comprises IDH-mutant astrocytoma, IDH-mutant and 1p/19q-codeleted oligodendroglioma, and IDH-wildtype glioblastoma according to the 2021 World Health Organization (WHO) Classification of Tumors of the Central Nervous System (CNS) [45]. Although most primary brain tumors, including gliomas, occur sporadically, familial aggregation of primary brain tumors has been reported in approximately 5% of patients [47, 81]. A number of monogenic autosomal-dominant hereditary tumor syndromes, such as Lynch, Li-Fraumeni, and melanoma-astrocytoma syndrome, caused by heterozygous pathogenic germline variants in cancer predisposition genes (CPGs), such as *PMS2*, *TP53*, and *CDKN2A*, are associated with an increased risk of gliomas [34, 37]. Some autosomal-recessive tumor predisposition syndromes, such as ataxia telangiectasia, caused by biallelic variants in the *ATM* gene, have also been linked to glioma predisposition [37].

Recently, first attempts have been made to determine the prevalence and type of pathogenic germline variants in adult glioma using next-generation sequencing approaches, including targeted exome and whole-genome sequencing [15, 32, 51]. These studies investigated unselected glioma patients presenting at defined centers in the United States over a certain time period [32, 51], or glioma families with at least two members diagnosed with glioma each, recruited at 14 centers in the United States, Israel, Sweden, and Denmark [15], and identified known and novel glioma risk genes and variants therein. To further elucidate the genetic landscape of glioma predisposition, we performed whole-exome sequencing (WES) on leukocyte DNA of 213 adult glioma patients diagnosed and treated in Germany with a familial and/or personal medical history of tumors. Data were analyzed using a candidate gene approach taking established CPGs and suspected glioma risk genes into account (approach 1), and a comparison of genetic findings was done in patients and controls (approach 2). We present detailed genotype–phenotype data for each carrier of a pathogenic germline variant (GV) in CPGs and glioma risk genes and the respective family, explore the resulting pathomechanisms of glioma tumorigenesis, and discuss potential therapeutic options for GV carrying patients.

## Materials and methods

### Human samples

The study was approved by the ethics boards of the participating centers in Hannover, Germany. Each family provided informed consent for participation in the study. The glioma cohort consisted of 206 families with at least one glioma patient each and presumed tumor predisposition due to (i) at least one additional glioma patient in the family (glioma family, *n* = 22, 11%), (ii) at least one additional brain tumor patient not otherwise specified in the family (brain tumor family, *n* = 36, 17%), (iii) at least one additional non-brain tumor patient in the family (tumor family, *n* = 107, 52%), or (iv) a glioma patient with a personal medical history of at least one syn- or metachronous non-brain tumor with an unremarkable family history (multiple tumors, *n* = 41, 20%). In the 206 families, blood samples were available from 213 patients diagnosed with a primary glioma at a median age of 58 years, who underwent brain tumor surgery during the time period December 2012–February 2024, mostly in Hannover, Germany. Tumor classification was originally done according to the WHO Classification of Tumours of the CNS of 2007, 2016, or 2021. In this study, the 213 primary gliomas were divided into four tumor types based on the WHO Classification of Tumours of the CNS of 2021: glioblastoma, IDH-wildtype (*n* = 112, 53%), astrocytoma, IDH-mutant (*n* = 33, 15%), oligodendroglioma, IDH-mutant and 1p/19q-codeleted (*n* = 25, 12%), or, if they did not fit unequivocally into the former three groups, as other gliomas (*n* = 43, 20%).

The control cohort used in approach 2 consisted of 391 in-house control individuals with no personal tumor history. These samples were chosen because they were from non-tumor individuals recruited at the same center and during the same time period as the glioma cohort, and sequencing and processing were identical. Control samples underwent WES concurrently with glioma samples and were analyzed in an identical manner.

### DNA extraction and WES

DNA was isolated from peripheral blood using the QIAamp DNA Blood Maxi Kit (Qiagen, Hilden, Germany) and from formalin-fixed paraffin-embedded (FFPE) glioma tissue using the QIAamp DNA FFPE Advanced Kit (Qiagen). WES was performed on leukocyte DNA of 213 glioma patients and 391 control individuals, and on tumor DNA of five glioma patients carrying *BRCA2* or *MUTYH* GVs using the SureSelectXT Human All Exon (Agilent, Santa Clara, CA, USA) or IDT xGen Exome Research Panel v2 (Integrated DNA Technologies, Coralville, IA, USA) target enrichment kit on a HiSeq or NovaSeq sequencer (all Illumina, San Diego, CA, USA). All samples were sequenced to a mean target coverage of ≥ 50x (leukocyte DNA) or > 200x (tumor DNA).

### WES data analysis

Sequencing data were aligned to the human reference genome build hg38/GRCh38, and variations were called using CLC Genomic Workbench (version 24.0.2; Qiagen). Variations were annotated and prioritized using Clinical Insight Interpret Translational (Qiagen). Quality filters were applied (coverage ≥ 20, call quality ≥ 25, allele fraction ≥ 30) and non-silent variants (splice site region up to 5 bases into intron, frameshift, in-frame indel (only in approach 1), stop gain/loss, and non-synonymous missense variants) were retained. Variant minor allele frequencies (MAF) for the non-Finnish European population were retrieved from the Genome Aggregation Database browser v4.1.0 (https://gnomad.broadinstitute.org). For prediction of variant deleteriousness, the tools CADD (https://cadd.gs.washington.edu), SIFT (https://sift.bii.a-star.edu.sg), PolyPhen-2 (http://genetics.bwh.harvard.edu/pph2), FATHMM Cancer (http://fathmm.biocompute.org.uk/cancer.html), and the metapredictor REVEL were used. An effect of variants on splicing was predicted by SpliceAI (https://spliceailookup.broadinstitute.org/) and MaxEntScan (https://github.com/matthdsm/MaxEntScan). Clinical classification of variants was retrieved from the ClinVar database (https://www.ncbi.nlm.nih.gov/clinvar).

**Fig. 1:**
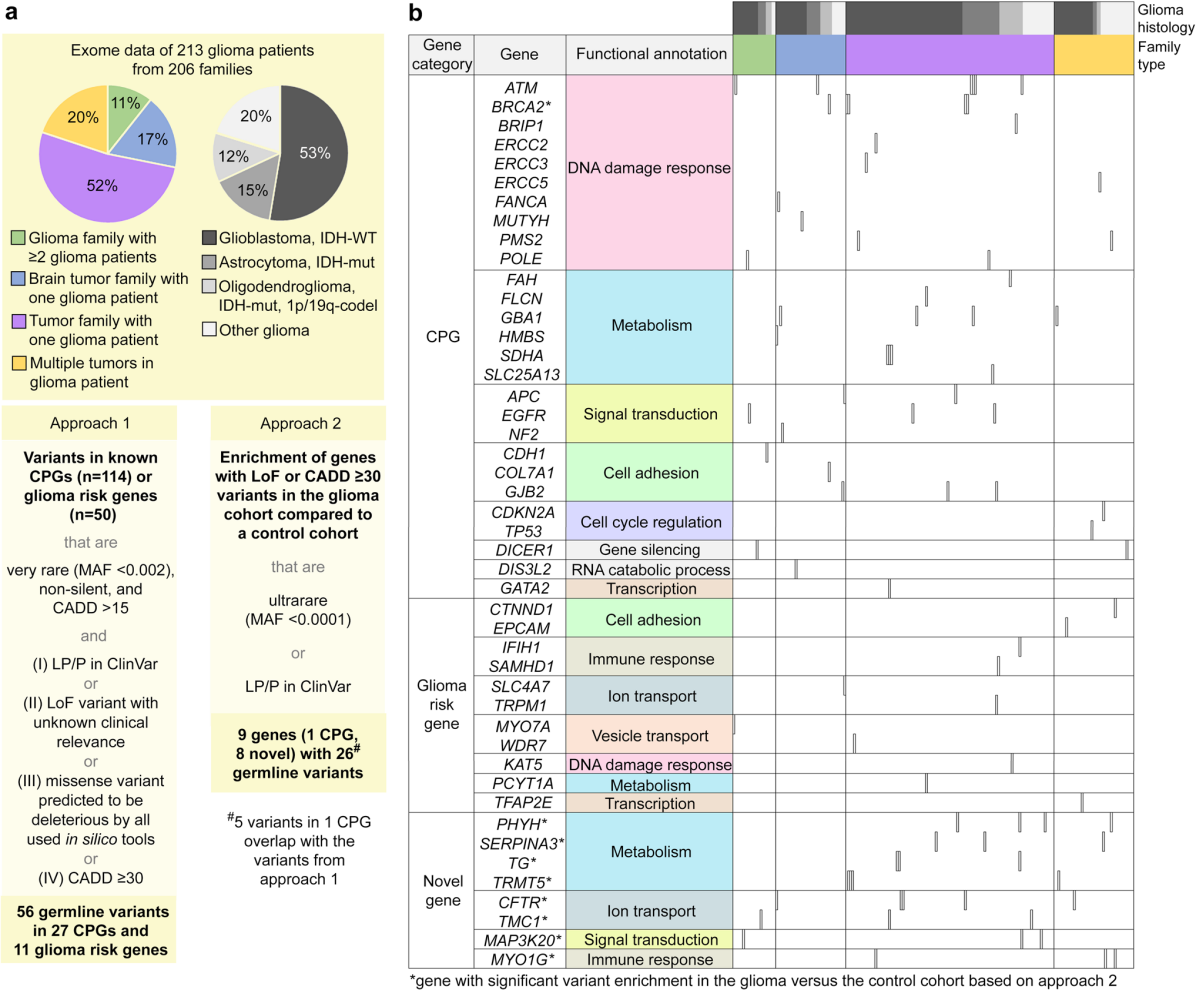
Whole-exome sequencing to identify genetic determinants of glioma predisposition: study design with an overview of diagnostic yield, and landscape of pathogenic germline variants and affected genes. **a** Scheme describing the family type and glioma histology of the study cohort and the two approaches used for analysis of WES data with an overview of the diagnostic yield. **b** Landscape of pathogenic germline variants in CPGs, suspected glioma risk genes, and novel candidate genes sorted by gene category, functional annotation, and family type. Glioma histology is also indicated. *CADD* combined annotation dependent depletion score; *ClinVar* ClinVar database; *codel* codeleted; *CPG* cancer predisposition gene; *IDH* isocitrate dehydrogenase 1/2; *LoF* loss-of-function; *LP* likely pathogenic; *MAF* minor allele frequency; *mut* mutant; *P* pathogenic; *WT* wildtype

Two approaches were used to prioritize the identified GVs. In approach 1, we only considered GVs in 114 established CPGs according to Rahman 2014 [63], and 50 potential glioma risk genes according to Choi et al. [15] and others (genes and references are listed in Supplementary Table 1 online resource) that were very rare (MAF < 0.002), had a CADD score > 15, and were (i) likely pathogenic or pathogenic (LP/P) according to the ClinVar database, (ii) predicted loss-of-function (LoF) variants with conflicting pathogenicity in ClinVar or not listed, (iii) missense variants predicted to be deleterious by all in silico tools used (CADD, SIFT, PolyPhen-2, REVEL, and FATHMM Cancer), or (iv) had a high CADD score (≥ 30), but did not fulfill criteria (i), (ii) or (iii) (Fig. 1a). For these GVs, the term pathogenic GVs was used. In approach 2, we identified genes with an enrichment of LoF variants (including start-loss, frameshift, splice region (± 5) with an effect on splicing according to MaxEntScan, and stop-gain variants, excluding in-frame indels) and/or of non-silent variants with a CADD score ≥ 30 (equivalent to the top 0.1% most deleterious variants) that were ultrarare (MAF < 0.0001) or classified as LP/P (ClinVar, any level of evidence) in the germline of the glioma (*n* = 206 families) compared to the control (*n* = 391 families) cohort. Only unrelated genetic data, i.e., from one glioma patient per family, were used, and only genes with no variants specified above in the control cohort and ≥ 3 variants specified above in the glioma cohort were considered in approach 2. Genes with GVs identified by approach 1 and 2 were functionally annotated using the web tool DAVID (https://davidbioinformatics.nih.gov).

### Expression analysis in glioma sections by immunohistochemistry

Immunohistochemical staining of ATRX, IDH1 R132H, and p53 was done as previously described [30]. The expression of ATM, BRCA2, phospho-H2AX, RAD51, and PARP1 was analyzed on FFPE glioma sections from patients with GVs in *ATM*, *BRCA2*, *FANCA*, *SDHA* after heat-induced epitope retrieval in 10 mM citrate buffer pH 6.0. The primary antibodies used were: rabbit anti-ATM (1:40 dilution; ab32420, Abcam, Cambridge, United Kingdom), mouse anti-BRCA2 (1:40 dilution; MAB2476, R&D Systems, Inc., Minneapolis, MN, USA), rabbit anti-phospho-H2AX (Ser139) (1:100 dilution; #9718, Cell Signaling Technologies, Danvers, MA, USA), rabbit anti-RAD51 (1:400 dilution; GTX100469, GeneTex, Irvine, CA, USA), and rabbit anti-poly(ADP-ribose) polymerase 1 (PARP1) (1:50 dilution; ab6079, Abcam). As secondary antibodies, horseradish peroxidase-labeled donkey anti-mouse or anti-rabbit IgG (1:300 dilution; A16017 / A16035, Thermo Fisher Scientific, Waltham, MA, USA) were used. Sections were counterstained with Mayer’s hemalum solution (Carl Roth GmbH + Co. KG, Karlsruhe, Germany), scanned using the Metafer Scanning Platform (MetaSystems Hard & Software, Altlussheim, Germany), and digital images were processed using ImageScope v11.2.0.780 software (Leica Microsystems, Wetzlar, Germany). The nuclear staining was assessed in five non-overlapping fields per section, and the immunoreactivity score (IRS) was determined. The IRS is the product of a proportion score (0: 0%, 1: < 10%, 2: 10–50%, 3: 50–80%, 4: > 80% positive cells) and an intensity score (0: negative, 1: weak, 2: moderate, 3: strong reaction) with a range from 0 to 12. The mean IRS was calculated from five fields per section (0–1: negative, 2–3: weak, 4–8: moderate, 9–12: strong expression).

The expression of EGFR and phospho-EGFR (Tyr1068) was analyzed on FFPE tumor sections from glioblastoma patients with GVs in *EGFR* after heat-induced epitope retrieval in Cell Conditioning 1 (Roche, Basel, Switzerland) or in 10 mM citrate buffer pH 6.0. The primary antibodies used were: rabbit anti-EGFR (undiluted, 0.4 µg/ml; 790–4347, Roche) and rabbit anti-phospho-EGFR (Tyr1068) (1:100 dilution; #3777, Cell Signaling Technologies). As secondary antibodies, ultraView Universal HRP Multimer (undiluted; included in the ultraView Universal DAB Detection Kit, 760–500, Roche) or horseradish peroxidase-labeled donkey anti-rabbit IgG (1:300 dilution; A16035, Thermo Fisher Scientific) were used. Sections were counterstained with Mayer’s hemalum solution (Carl Roth GmbH + Co. KG). For histological evaluation, consecutive sections were stained with hematoxylin-eosin according to standard protocols. Images were acquired using a BX46 microscope and a XC50 camera (all Olympus, Shinjuku, Japan).

### Molecular glioma characterization

Variant hotspot sequencing of the *IDH1* and *IDH2* genes was done, and *MGMT* promoter methylation was determined according to previously reported protocols [16]. Fluorescence in situ hybridization of the *EGFR* locus, variant hotspot sequencing of the *H3F3A*, *HIST1H3B*, and *TERT* genes, and digital PCR to assess *CDKN2A/B* deletion were reported in detail elsewhere [30]. To determine the tumor mutational burden (TMB), glioma tissue was microdissected from 5 µm FFPE tumor sections. DNA was extracted using the Maxwell RSC DNA FFPE Kit (Promega, Madison, WI, USA) and a Maxwell RSC Instrument (Promega). For TMB detection, 15 ng tumor DNA was used in an Oncomine Tumor Mutation Load Assay (*n* = 409 genes, 1.7 Mb exonic coverage, Thermo Fisher Scientific) on an Ion S5 Prime System (Life Technologies, Carlsbad, CA, USA). For TMB calculation, quality and population filters were applied (coverage ≥ 60, strand bias ≥ 0.9, MAF < 0.05). Second hit analysis was based on WES data obtained from tumor DNA of glioma patients carrying *BRCA2* or *MUTYH* GVs, taking variants with a MAF < 0.01 into account.

### Statistical analysis

Statistical analysis was conducted using MATLAB (The MathWorks, Natick, MA, USA) or Prism software (GraphPad Software, Boston, MA, USA) and Mann–Whitney test or Fisher’s exact test (both two-tailed). *P* values < 0.05 were considered significant. Multiple testing in approach 2 was adjusted for with a false discovery rate of 10% according to Benjamini, Krieger, and Yekutieli.

## Results

### Rare deleterious variants in CPGs or suspected glioma risk genes in the germline of glioma patients with presumed tumor predisposition

To explore the role of GVs in glioma risk, we analyzed germline WES data of 213 glioma patients from 206 families with evidence for a tumor predisposition using two approaches (Fig. 1a). In approach 1, we prioritized very rare (MAF < 0.002) GVs with a CADD score > 15 in 114 established CPGs [24, 63, 80] and 50 potential glioma risk genes identified by us or others [3, 6, 15, 18] (genes and references listed in Supplementary Table 1 online resource). Of the detected 348 GVs, 20 were ClinVar LP/P, 10 were predicted LoF variants with conflicting pathogenicity in ClinVar or not listed, 18 were missense variants predicted to be deleterious by all in silico tools used (CADD, SIFT, PolyPhen-2, REVEL, FATHMM Cancer), and eight had a high CADD score (≥ 30), but did not fulfill the other criteria (Table 1). Taken together, 56 GVs in 27 CPGs and 11 suspected glioma risk genes were considered pathogenic GVs (Fig. 1a), and 56 glioma patients from 54 families were affected by at least one pathogenic GV in a CPG and/or glioma risk gene (Supplementary Tables 2a, 2b online resource). The CPGs affected by pathogenic GVs were most frequently associated with DNA damage response (e.g., *ATM*, *BRCA2*, *PMS2*, *POLE*), metabolism (e.g., *GBA1*, *SDHA*), signal transduction (e.g., *APC*, *EGFR*), and cell adhesion (e.g., *CDH1*, *GJB2*) (Fig. 1b). Potential glioma risk genes with a pathogenic GV most frequently played roles in cell adhesion (*CTNND1*, *EPCAM*), immune response (*IFIH1*, *SAMHD1*), and ion transport (*SLC4A7*, *TRPM1*) (Fig. 1b).

**Table 1:** Heterozygous germline variants identified in cancer predisposition genes or suspected glioma risk genes in 213 adult glioma patients with presumed tumor predisposition from 206 families using approach 1

Patient ID	Gene	Inheritance^a^	AD inheritance previously observed^b^	Genomic position (GRCh38/hg38)	Nucleotide change	Protein/molecular consequence	Clin Var^c^	MAF^d^(%)	Prediction according to CADD^e^/SIFT^f^/PolyPhen-2^ g^/REVEL^h^/FATHMM Cancer^i^/SpliceAI^j^
Likely pathogenic or pathogenic variants according to ClinVar									
WI207-III.1	*ATM*	AD/AR	+	11:108,331,877	c.7630-2A>C	Splice acceptor	P	0.001441	34.0/—/—/—/—/0.91
WI166-III.1	*ATM*	AD/AR	+	11:108,332,848	c.7875_7876delinsGC	p.(D2625_A2626delinsEP)	LP/P	0	22.4/—/—/—/—/—
WI14-III.1	*BRCA2*	AD/AR	+	13:32,319,330	c.316+5G>C	Splice region	P	0	22.3/—/—/—/—/0.92
WI60-III.1	*BRCA2*	AD/AR	+	13:32,336,579	c.2224C>T	p.(Q742*)	P	0	33.0/—/—/—/—/—
WI226-III.1	*BRCA2*	AD/AR	+	13:32,337,185	c.2830A>T	p.(K944*)	P	0.000339	32.0/—/—/—/—/—
WI175-III.1	*BRCA2*	AD/AR	+	13:32,338,763	c.4409_4410del	p.(I1470Kfs*11)	P	0	21.2/—/—/—/—/—
WI86-III.1	*BRIP1*	AD/AR	+	17:61,859,791	c.205+5G>T	Splice region	LP	0	24.2/—/—/—/—/0.85
WI04-III.1	*CDKN2A*	AD	+	9:21,971,200	c.159G>A	p.(M53I)	LP/P	0	27.1/D/B/0.724/−2.86/0.02
WI191-III.1	*COL7A1*	AD/AR	+	3:48,570,639	c.7344G>A	p.(V2448V) splice donor	P	0.006638	22.8/—/—/—/—/0.63
WI165-III.1	*EPCAM*	AD/AR	+	2:47,374,035	c.412C>T	p.(R138*)	P	0.0001695	42.0/—/—/—/—/—
WI50-III.1	*FAH*	AR	−	15:80,180,230	c.1062+5G>A	Splice region	P	0.04642	23.5/—/—/—/—/ 0.78
WI209-III.1	*FANCA*	AR	(+)^k,l^	16:89,746,848	c.3391A>G	p.(T1131A)	LP/P	0.01614	25.5/D/PoD/0.842/3.74/0.09
WI166-III.1 WI205-III.1 WI214-III.1	*GBA1*	AD/AR	−	1:155,235,843	c.1226A>G	p.(N409S)	LP/P	0.1728	24.1/D/B/0.673/2.36/0.21
WI09-III.1	*GBA1*	AD/AR	−	1:155,235,195	c.1505G>A	p.(R502H)	LP/P	0.0003391	34.0/D/PrD/0.842/2.32/0.38
WI75-III.1	*GJB2*	AD/AR	+	13:20,189,117	c.465T>A	p.(Y155*)	P	0.00008474	29.5/—/—/—/—/—
WI48-III.1 WI49-III.1	*GJB2*	AD/AR	+	13:20,189,473	c.109G>A	p.(V37I)	P	0.08816	21.7/T/PrD/0.656/3.08/0.02
WI70-III.1	*MUTYH*	AR	(+)^m,n^	1:45,332,445	c.650G>A	p.(R217H)	LP/P	0.006695	25.8/D/PrD/0.927/0.27/0.08
Fam003-III.1	*MYO7A*	AD/AR	(+)^o^	11:77,184,688	c.3476G>T	p.(G1159V)	LP/P	0.05355	29.1/D/PrD/0.938/0.53/0.00
WI78-III.1	*SAMHD1*	AD/AR	−	20:36,951,576	c.68C>G	p.(S23*)	LP/P	0.002542	33.0/—/—/—/—/—
LI06-III.1 WI87-III.1	*SDHA*	AD/AR	+	5:223,509	c.91C>T	p.(R31*)	LP/P	0.05341	34.0/—/—/—/—/—
Predicted loss-of-function variants with conflicting pathogenicity in ClinVar or not listed									
WI105-III.1	*ATM*	AD/AR	+	11:108,245,028	c.901+2T>A	Splice donor	–	0	33.0/—/—/—/—/0.99
WI160-III.1	*ATM*	AD/AR	+	11:108,365,366	c.9029T>G	p.(L3010*)	–	0	42.0/—/—/—/—/—
WI191-III.1	*BRCA2*	AD/AR	+	13:32,398,608	c.10095delinsGAATTATATCT	p.(S3366Nfs*4)	C	0	24.2/—/—/—/—/—
WI72-III.1	*ERCC5*	AR	−	13:102,854,319	c.412C>T	p.(R138*)	–	0.000339	36.0/—/—/—/—/—
WI53-III.1	*KAT5*	AD	−	11:65,718,667	c.1342C>T	p.(R448*)	–	0.0001695	37.0/—/—/—/—/—
WI222-III.1	*NF2*	AD	+	22:29,694,776	c.1762C>T	p.(R588*)	VUS	0.000678	40.0/—/—/—/—/—
WI22-III.2	*PCYT1A*	AR	−	3:196,238,789	c.1003C>T	p.(R335*)	VUS	0.001028	40.0/—/—/—/—/—
WI88-III.1	*POLE*	AD/AR	+	12:132,687,315	c.1A>T (start codon variant)	p.(M1L)	C	0.01495	21.9/D/B/0.257/3.37/0.01
WI183-III.1	*SDHA*	AD/AR	+	5:254,390	c.1795-3C>G	Splice region	C	0.0001706	24.1/—/—/—/—/0.85
WI48-III.1	*TRPM1*	AR	(+)^p^	15:31,002,524	c.4173_4176delAGAC	p.(D1392Lfs*11)	VUS	0.01669	25.0/—/—/—/—/—
Missense variants with pathogenic predictions in all used in silico tools^q^									
WI99-III.1	*APC*	AD	+	5:112,841,473	c.5879C>T	p.(P1960L)	C	0.005341	25.4/D/PrD/0.878/−5.39/0.00
WI89-III.1	*APC*	AD	+	5:112,842,085	c.6491G>T	p.(G2164V)	–	0	26.2/D/PrD/0.558/−4.46/0.00
WI37-III.1	*ATM*	AD/AR	+	11:108,310,267	c.5870A>G	p.(Y1957C)	VUS	0.0001695	26.9/D/PrD/0.602/−2.22/0.01
Fam004-III.1	*ATM*	AD/AR	+	11:108,326,070	c.6820G>A	p.(A2274T)	C	0.01822	27.5/D/PoD/0.524/−0.94/0.02
Fam002^r^ -II.2 -III.1, -III.2	*CDH1*	AD	+	16:68,833,300	c.2450C>T	p.(A817V)	C	0.002119	27.2/D/PrD/0.574/−1.22/0.03
WI239-III.1	*DICER1*	AD	+	14:95,091,226	c.5504A>G	p.(Y1835C)	C	0.004576	28.8/D/PoD/0.736/−1.05/0.15
WI61-III.1	*DICER1*	AD	+	14:95,107,993	c.2537T>G	p.(I846S)	–	0	24.5/D/PoD/0.739/−1.63/0.07
WI201-III.1	*EGFR*	AD/AR	+	7:55,154,015	c.752G>C	p.(C251S)	–	0	27.7/D/PrD/0.876/−3.48/0.02
WI177-II.1	*EGFR*	AD/AR	+	7:55,174,726	c.2189T>G	p.(L730R)	C	0.002204	31.0/D/PrD/0.573/−1.9/0.02
WI33-III.1	*EGFR*	AD/AR	+	7:55,200,352	c.2885G>A	p.(R962H)	C	0.02229	29.5/D/PoD/0.543/−1.97/0.07
WI153-III.1	*ERCC2*	AR	(+)^s^	19:45,352,511	c.2041G>A	p.(D681N)	C	0.001695	24.9/D/PrD/0.898/−1.48/0.05
WI22-III.2	*FLCN*	AD	+	17:17,216,464	c.1216A>G	p.(S406G)	C	0.004407	25.3/D/PoD/0.672/−2.08/0.04
LI06-III.1	*GATA2*	AD	+	3:128,487,002	c.30G>T	p.(W10C)	C	0.01881	32.0/D/PoD/0.947/−1.25/0.00
WI169-II.2	*HMBS*	AD/AR	+	11:119,088,642	c.95G>A	p.(R32H)	VUS	0.0001696	29.2/D/PrD/0.885/NA/0.06
WI11-III.1	*PMS2*	AD/AR	+	7:5,992,044	c.917T>C	p.(V306A)	VUS	0.001916	27.6/D/PrD/0.824/−1.66/0.00
WI103-III.1	*PMS2*	AD/AR	+	7:5,995,563	c.874A>T	p.(I292F)	VUS	0.0002543	25.7/D/PrD/0.866/−1.88/0.06
WI51-III.1	*TFAP2E*	–	(+)^t^	1:35,590,037	c.893C>T	p.(S298L)	VUS	0.008984	28.3/D/PoD/0.922/−1.28/0.01
WI236-III.1	*TP53*	AD	+	17:7,675,220	c.392A>G	p.(N131S)	VUS	0.00008475	27.0/D/PrD/0.859/−9.14/0.00
Variants with a CADD score ≥ 30, but not fulfilling the other criteria									
WI122-III.1	*CTNND1*	AD	(+)^u^	11:57,814,325	c.2653C>T	p.(R885W)	VUS	0.001867	32.0/D/PoD/0.414/2.28/0.12
WI163-III.1	*DIS3L2*	AR	(+)^v^	2:232,136,590	c.821G>A	p.(R274Q)	VUS	0	31.0/D/PrD/0.497/2.45/0.03
WI126-II.2	*ERCC3*	AR	(+)^w^	2:127,286,825	c.1220T>G	p.(I407S)	–	0	30.0/D/PrD/0.710/1.87/0.00
WI106-III.1	*IFIH1*	AD/AR	−	2:162,277,667	c.1792C>T	p.(R598C)	VUS	0.003148	32.0/D/PrD/0.556/2.27/0.00
Fam016-III.1	*POLE*	AD/AR	+	12:132,657,402	c.3406C>T	p.(R1136W)	VUS	0.0004237	30.0/D/PrD/0.598/0.57/0.00
WI89-III.1	*SLC4A7*	–	−	3:27,394,756	c.2879A>G	p.(Y960C)	–	0.0004238	32.0/D/PrD/0.939/3.01/0.00
WI145-III.1	*SLC25A13*	AR	(+)^x^	7:96,189,581	c.848G>A	p.(G283E)	VUS	0.009086	33.0/D/B/0.709/3.42/0.72
WI08-III.1	*WDR7*	–	−	18:56,816,031	c.3191C>T	p.(P1064L)	VUS	0.0005124	33.0/D/PoD/0.242/0.01/0.00

Listed are very rare (MAF < 0.002), non-silent (i.e., splice site, frameshift, in-frame indels, stop gain/loss and non-synonymous missense) variants with a CADD score > 15, classified as LP/P in ClinVar, predicted loss-of-function variants with conflicting pathogenicity in ClinVar or not listed, missense variants with pathogenic predictions in all used in silico tools, or with a CADD score ≥ 30, but not fulfilling the other criteria

*AD* autosomal dominant; *AR* autosomal recessive; *C* conflicting classifications of pathogenicity in the ClinVar database; *delins* deletion insertion; *D* damaging; *fs* frameshift; *LP* likely pathogenic; *MAF* minor allele frequency; *P* pathogenic; *PoD* possibly damaging; *PrD* probably damaging; *T* tolerated; *VUS* variant of uncertain significance; *, stop gain; –, no data

^a^Inheritance according to the OMIM database (https://www.omim.org)

^b^Autosomal dominant inheritance observed in cancer patients according to Rahman 2014 [63] (indicated by +) or the reports cited in the footnotes

^c^Classification according to the ClinVar database (www.ncbi.nlm.nih.gov/clinvar)

^d^Minor allele frequency according to the Genome Aggregation Database (gnomAD) browser version 4.1.0, non-Finnish European population (https://gnomad.broadinstitute.org)

^e^According to CADD (https://cadd.gs.washington.edu)

^f^According to SIFT (https://sift.bii.a-star.edu.sg)

^g^According to PolyPhen-2 (http://genetics.bwh.harvard.edu/pph2)

^h^According to REVEL (https://sites.google.com/site/revelgenomics/downloads/revel-genome-segment-files)

^i^According to Functional Analysis through Hidden Markov Models (FATHMM) version v2.3 for cancer-promoting/driver mutations and other germline polymorphisms (http://fathmm.biocompute.org.uk/cancer.html)

^j^According to SpliceAI (https://spliceailookup.broadinstitute.org)

^k^Autosomal dominant inheritance observed in cancer patients[71]

^l^Autosomal dominant inheritance observed in cancer patients[1]

^m^Autosomal dominant inheritance observed in cancer patients[4]

^n^Autosomal dominant inheritance observed in cancer patients[36]

^o^Autosomal dominant inheritance observed in cancer patients[23]

^p^Autosomal dominant inheritance observed in cancer patients[79]

^q^Variants with CADD score ≥ 20; FATHMM Cancer ≤ −0.75; REVEL score > 0.5; predicted to be damaging in SIFT and PolyPhen-2

^r^This family was previously published[24]

^s^Autosomal dominant inheritance observed in cancer patients[82]

^t^Autosomal dominant inheritance observed in cancer patients[12]

^u^Autosomal dominant inheritance observed in cancer patients[29]

^v^Autosomal dominant inheritance observed in cancer patients[25]

^w^Autosomal dominant inheritance observed in cancer patients[74]

^x^Autosomal dominant inheritance observed in cancer patients[14]

### Genes enriched with LoF or deleterious missense GVs in glioma patients with presumed tumor predisposition compared to a control cohort

In approach 2, we searched for genes significantly more frequently affected by LoF variants (excluding in-frame indels) or missense variants with a CADD score ≥ 30 that were ultrarare (MAF < 0.0001) or ClinVar LP/P in the glioma cohort, i.e., glioma index patients from 206 families, versus a control cohort, i.e., control individuals from 391 families. After adjustment for multiple testing with a false discovery rate of 10%, a significant GV enrichment in the glioma versus the control cohort was found for nine genes, i.e., one CPG (*BRCA2*) and eight novel genes not previously associated with glioma risk, with 26 different GVs considered pathogenic GVs (Fig. 1a, Supplementary Table 3 online resource). Pathogenic GVs in *BRCA2* were detected in five families of the glioma cohort but not in controls (*P* = 0.005), implicating *BRCA2* using both approaches utilized here. The eight novel genes with pathogenic GVs in three to five families from the glioma cohort but not in controls (*P* < 0.05) play roles in metabolism (*PHYH*, *SERPINA3*, *TG*, *TRMT5*), ion transport (*CFTR*, *TMC1*), immune response (*MYO1G*), and signal transduction (*MAP3K20*) (Fig. 1b, Supplementary Table 3 online resource).

### All genetic determinants of glioma predisposition identified in glioma patients with presumed tumor predisposition

**Fig. 2:**
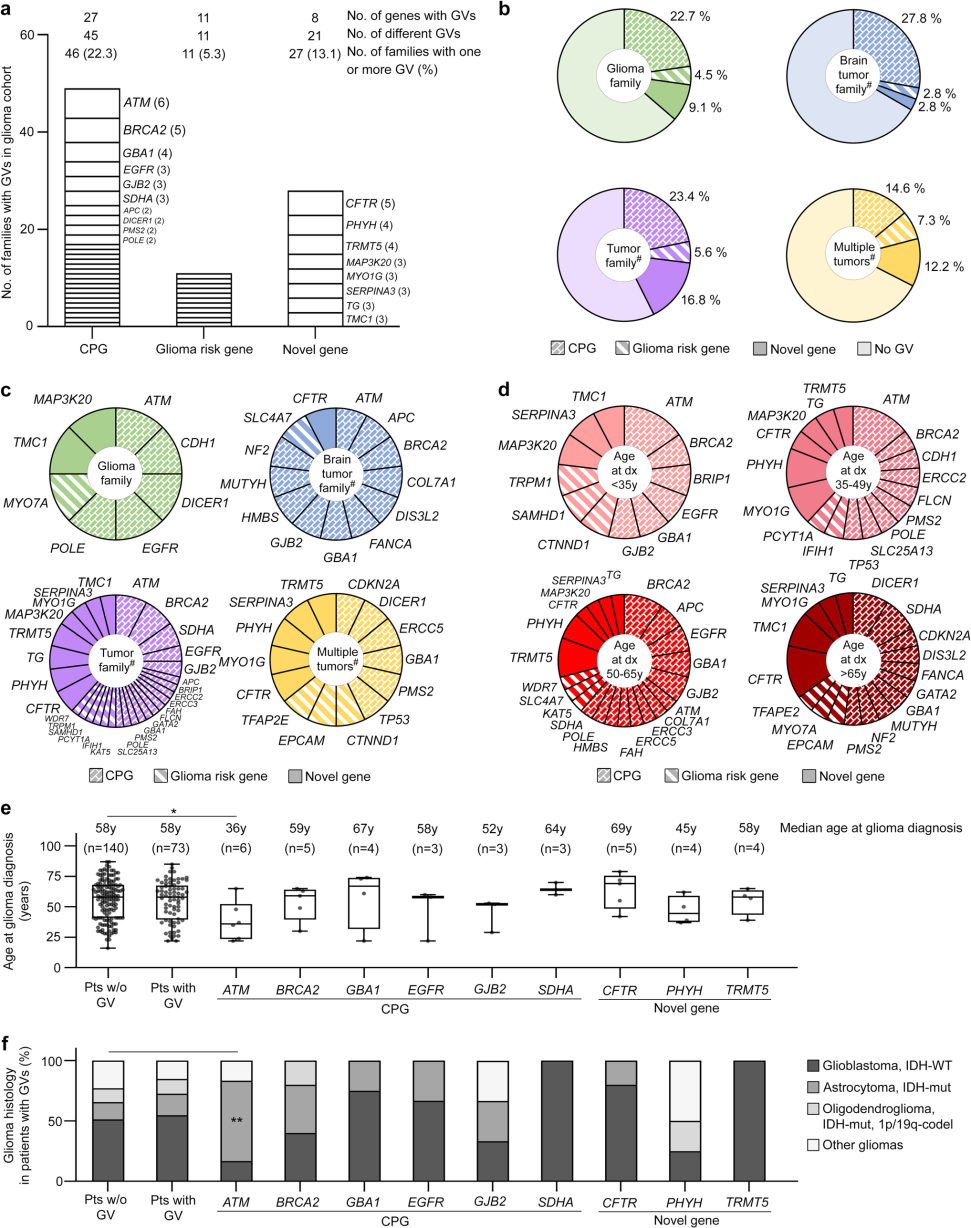
Whole-exome sequencing results in 213 glioma patients with a familial and/or personal history of tumors from 206 families obtained by approach 1 and 2, and characteristics of 73 glioma patients with pathogenic GVs. **a** Number of glioma families with pathogenic GVs in CPGs, suspected glioma risk genes, and novel candidate genes. Genes recurrently affected are specified, and the number of families with a GV in a certain gene is given in brackets. **b** Diagnostic yield of pathogenic GVs in CPGs, potential glioma risk genes, and novel candidate genes in the four types of families with one glioma patient each and at least another glioma patient (glioma family), brain tumor patient (brain tumor family), tumor patient (tumor family) or no family history of tumors but syn-/metachronous non-brain tumors in the personal history of the glioma patient (multiple tumors). **c** Genes (CPGs, suspected glioma risk genes, and novel candidate genes) affected by pathogenic GVs in the four types of families with at least one glioma patient each. In the pie chart referring to tumor families, the size of each segment reflects the number of families with a GV in the specified gene. **d** Genes (CPGs, suspected glioma risk genes, and novel candidate genes) affected by pathogenic GVs in four age groups. In the pie charts, the size of each segment reflects the number of families with a GV in the specified gene. **e** Age at glioma diagnosis of patients without GVs, with GVs, and with GVs in specific genes. Shown are box plots and data points. The median age at glioma diagnosis of *ATM* GV carriers was significantly lower than that of patients without GVs. **f** Glioma histology of patients without GVs, with GVs, and with GVs in specific genes. *ATM* GV carriers were significantly more frequently diagnosed with IDH-mutant astrocytoma than patients without GVs. ^#^, each family contains one glioma patient. *Codel* codeleted; *CPG* cancer predisposition gene; *dx* diagnosis of primary glioma; *GV* germline variant; *IDH* isocitrate dehydrogenase 1/2; *multiple tumors* glioma patient with syn-/metachronous non-brain tumor(s) in personal medical history and unremarkable family history; *mut* mutant; *No.* number; *Pts* patients; *w/o* without; *WT* wildtype; *y* years. *, *P* < 0.05 (two-tailed Mann–Whitney test); **, *P* < 0.01 (two-tailed Fisher’s exact test)

When combining the results from approach 1 (Table 1) and from approach 2 (Supplementary Table 3 online resource), CPGs were affected by at least one pathogenic GV in 46/206 (22.3%) families, suspected glioma risk genes in 11/206 (5.3%) families, and novel genes with significant GV enrichment in 27/206 (13.1%) families of the glioma cohort (Fig. 2a). The CPGs most frequently affected by GVs were *ATM* in 6/206 (2.9%) families, *BRCA2* in 5/206 (2.4%) families (identified in approaches 1 and 2), *GBA1* in 4/206 (1.9%) families as well as *EGFR, GJB2*, and *SDHA* in 3/206 (1.5%) families each of the glioma cohort (Fig. 2a). No potential glioma risk gene was recurrently affected by GVs. The novel genes most frequently affected by GVs were *CFTR* in 5/206 (2.4%) families followed by *PHYH* and *TRMT5* in 4/206 (1.9%) families each of the glioma cohort (Fig. 2a, Supplementary Table 3 online resource). While the fraction of pathogenic GVs in CPGs was similar in glioma families with at least two glioma patients each as well as in brain tumor families and tumor families with at least one glioma patient each (22.7%, 27.8%, and 23.4%), it was lower in multiple tumor glioma patients who had a personal medical history of at least one syn- or metachronous non-brain tumor but an unremarkable family history (14.6%) (Fig. 2b). Pathogenic GVs in *ATM* were found in each family type with the highest fraction in glioma families, but not in multiple tumor glioma patients (Fig. 2c). Pathogenic GVs in *BRCA2* were detected in tumor families and brain tumor families. Pathogenic GVs in suspected glioma risk genes and novel genes were observed in each family type (Fig. 2c).

### Age at glioma diagnosis and glioma histology of patients with pathogenic GVs

To compare the age at diagnosis of the primary glioma, the GV carriers were divided into four age groups (Fig. 2d). Five of six *ATM* GV carriers were diagnosed with a glioma before 50 years of age. *BRCA2* GV carriers were most frequently affected by glioma between the age of 50 and 65 years*.* The *DICER1* and most of the *SDHA* and *CFTR* GV carriers developed glioma after the age of 65 years (Fig. 2d). The median age at glioma diagnosis in GV carriers and in glioma patients without GVs was 58 years (*P* = 0.726; Fig. 2e). *ATM* GV carriers had a significantly younger median age at glioma diagnosis compared to non-GV carriers (36 versus 58 years, *P* = 0.022) (Fig. 2e).

Next, we compared the glioma histology in carriers of GVs in different genes and non-GV carriers. Astrocytoma, IDH-mutant was diagnosed significantly more frequently in glioma patients with *ATM* GVs than in those without GVs (4/6 versus 20/140, *P* = 0.007; Fig. 2f). Patients with GVs in *SDHA* (*n* = 3) or *TRMT5* (*n* = 4) were exclusively affected by glioblastoma, IDH-wildtype (Fig. 2f).

### Other tumors and familial tumor spectrum in glioma patients with pathogenic GVs

**Fig. 3:**
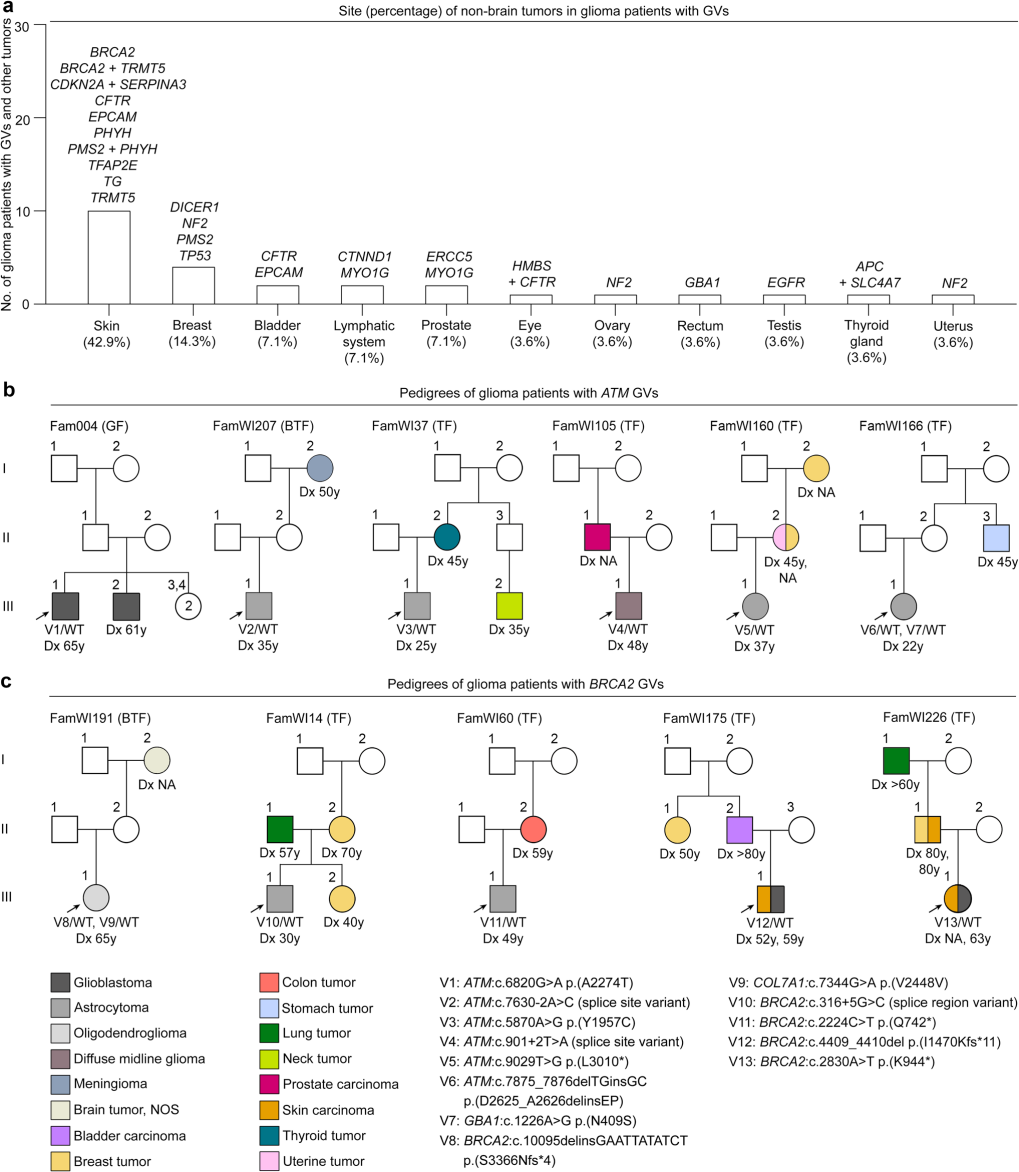
Spectrum of personal syn-/metachronous non-brain tumors and familial tumors in glioma patients with pathogenic GVs. **a** Number of glioma patients with GVs in the specified genes and syn- or metachronous non-brain tumors at the specified sites. **b**, **c** Familial tumor spectrum of glioma patients with *ATM* GVs (**b**) or *BRCA2* GVs (**c**), the most frequently affected CPGs in this study. Breast tumors were diagnosed in four first- or second-degree relatives of glioma patients with *BRCA2* GVs (**c**). The pedigrees of glioma patients with GVs in other CPGs and suspected glioma risk genes are shown in Supplementary Figs. 1–7 online resource. It is not specified whether an individual is alive or deceased. *BTF* brain tumor family with one glioma patient and at least another brain tumor patient; *CPG* cancer predisposition gene; *Dx* age at diagnosis of primary tumor; *GF* glioma family with at least two glioma patients; *GV* germline variant; *NA* not available; *No.* number; *NOS* not otherwise specified; *TF* tumor family with one glioma patient and at least another tumor patient; *V* variant; *WT* wildtype; *y* years

Syn- or metachronous tumors in other organs were diagnosed in 21/73 (28.8%) glioma patients with a pathogenic GV, recurrently affecting the skin, breast, bladder, lymphatic system, and prostate (Fig. 3, Supplementary Figs. 1–7 online resource, Supplementary Table 2a online resource). For example, two glioma patients with a *BRCA2* LoF GV also had skin tumors, and both glioma patients with a *PMS2* missense GV had a syn- or metachronous tumor of the skin or the breast (Fig. 3a, Fig. 3c, Supplementary Fig. 3 online resource). The carrier of an *EPCAM* LoF GV had two metachronous tumors affecting the skin and the bladder (Fig. 3a, Supplementary Fig. 4 online resource), and the carrier of an *NF2* LoF GV had three syn- or metachronous tumors affecting the breast, uterus, and ovary (Fig. 3a, Supplementary Fig. 6 online resource).

The tumor spectrum in the families of glioma patients with pathogenic GVs in CPGs or suspected glioma risk genes is shown in three-generation pedigrees (also indicating the age at tumor diagnosis, Fig. 3b-c, Supplementary Figs. 1–7 online resource) and specified in Supplementary Table 2a online resource. In *ATM* GV carriers and their families, tumors of the brain (8/15 tumors, 53%), breast (2/15, 13%), neck, prostate, stomach, thyroid, and uterus (1/15, 7% each) were diagnosed (Fig. 3b). The *ATM* GV carrier and his brother in family Fam004 were diagnosed with glioblastoma at almost the same age (61 and 65 years, Fig. 3b). The median age at tumor diagnosis in all affected family members of *ATM* GV carriers with available data (*n* = 12) was 45 years. In *BRCA2* GV carriers and their families, tumors of the brain (6/17, 35%), breast (4/17, 24%), skin (3/17, 18%), lung (2/17, 12%), bladder, and colon (1/17, 6% each) were observed (Fig. 3c). Breast tumors were diagnosed in first- or second-degree female or male relatives of glioma patients with *BRCA2* GVs in 3/5 families, strongly suggesting that the breast tumor patients also carry the *BRCA2* GV and that it is the tumor-predisposing variant in these families.

### Characterization of tumors from patients with GVs in CPGs associated with DNA damage response

**Fig. 4:**
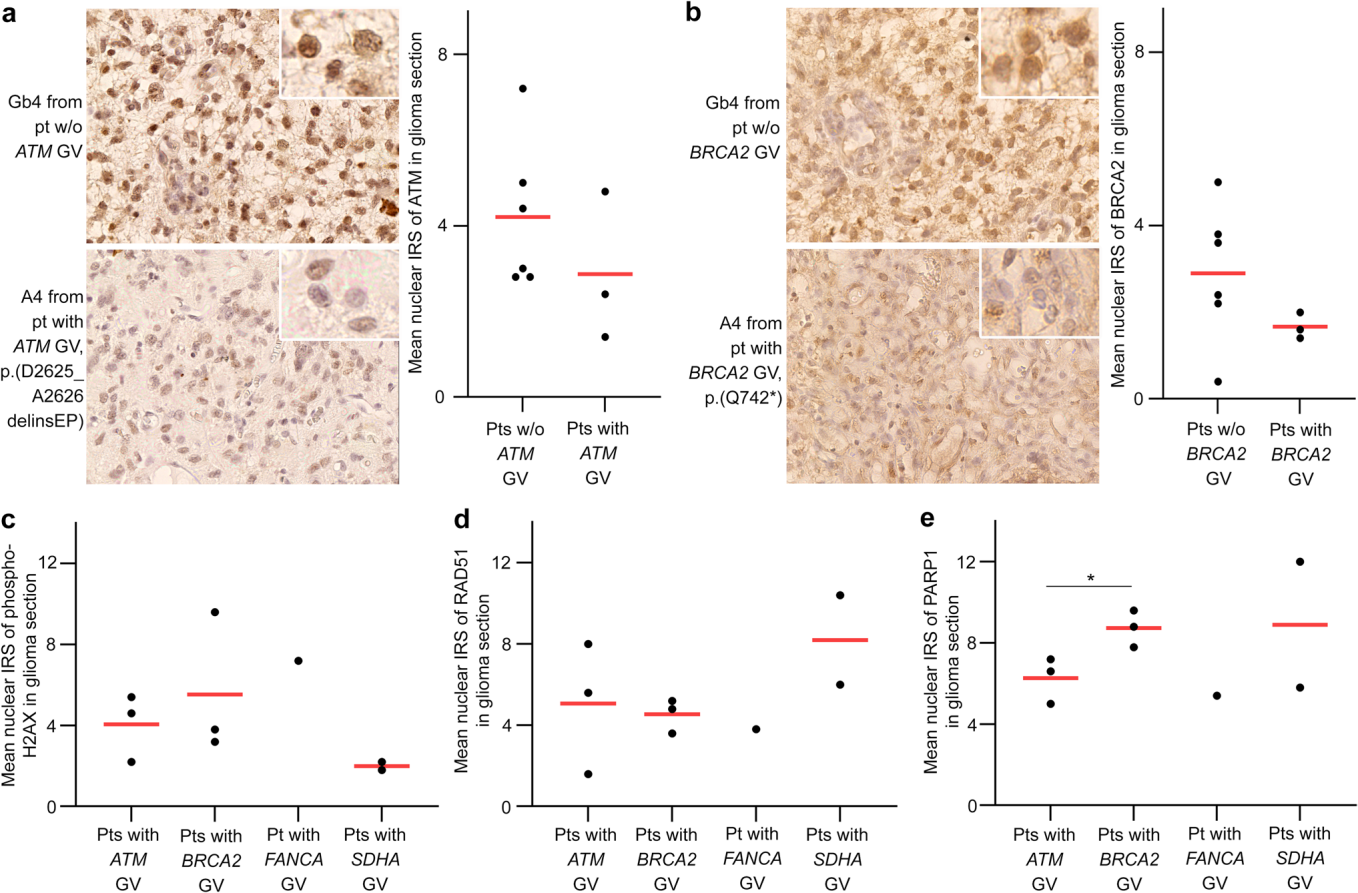
Characterization of FFPE glioma sections from patients with GVs in the CPGs *ATM*, *BRCA2*, *FANCA*, and *SDHA* directly or indirectly associated with DNA damage response by immunohistochemistry and semi-quantitative analysis of the nuclear IRS. **a**, **b** The mean IRS of nuclear ATM (**a**) or BRCA2 (**b**) expression was lower in patients with versus without GVs in *ATM* or *BRCA2*, respectively. **c**–**e** To assess DNA damage and DNA damage response, the nuclear expression of the markers for DNA DSBs (phospho-H2AX, **c**), homology-directed repair of DSBs (RAD51, **d**), and repair of DNA single-strand breaks (PARP1, **e**) was determined. *A4* astrocytoma, IDH-mutant, CNS WHO grade 4; *CNS* central nervous system; *CPG* cancer predisposition gene; *DSB* double-strand break; *FFPE* formalin-fixed paraffin-embedded; *Gb4* glioblastoma, IDH-wildtype, CNS WHO grade 4; *GV* germline variant; *IRS* immunoreactivity score; *pt* patient; *pts* patients; *WHO* World Health Organization; *w/o* without

DNA damage response genes were affected by 22/77 (28.6%) of the different pathogenic GVs (Figs. 1b, 2a). Therefore, we determined the expression of markers of DNA double-strand breaks (DSB, phospho-H2AX), homology-directed repair of DSB (RAD51), and repair of DNA single-strand breaks (PARP1) as well as the expression of *ATM* and *BRCA2* in available FFPE glioma sections from carriers of GVs in four genes conferring homologous recombination repair (HRR) deficiency, if mutated, i.e., *ATM* (*n* = 3 gliomas), *BRCA2* (*n* = 3 gliomas), *FANCA* (*n* = 1 glioma), and *SDHA* (*n* = 2 gliomas) (Supplementary Fig. 8 online resource). A semi-quantitative analysis of ATM and BRCA2 staining revealed a trend towards a lower mean nuclear IRS for ATM in gliomas from *ATM* GV versus non-*ATM* GV carriers, and for BRCA2 in gliomas from *BRCA2* GV versus non-*BRCA2* GV carriers (Fig. 4a, b). The mean nuclear IRS of phospho-H2AX indicating DSB accumulation was lowest in gliomas from *SDHA* versus *ATM*, *BRCA2*, and *FANCA* GV carriers, although the differences were not statistically significant (Fig. 4c), possibly because *SDHA* variants are least directly linked to impaired DSB repair. Gliomas from *SDHA* GV carriers with less DSB accumulation showed a trend towards a higher mean nuclear IRS of RAD51 than those from *ATM*, *BRCA2,* and *FANCA* GV carriers with more DSBs (Fig. 4c, d). The mean nuclear IRS of PARP1 was significantly higher in gliomas from *BRCA2* GV carriers than in those from *ATM* GV carriers (Fig. 4e).

Other characteristics of primary or recurrent gliomas of 56 patients affected by at least one pathogenic GV in a CPG and/or a suspected glioma risk gene determined by approach 1 are listed in Supplementary Table 2b online resource. In gliomas (*n* = 4) from patients with *MUTYH*, *PMS2*, or *POLE* GVs, the median TMB was elevated (7.28, range: 6.02–8.59 mutations per megabase, Supplementary Table 2b online resource) compared to that previously defined for gliomas (2.6 mutations per megabase) [78].

### Possible targeted therapy options for glioma patients with pathogenic GVs in certain CPGs

**Fig. 5:**
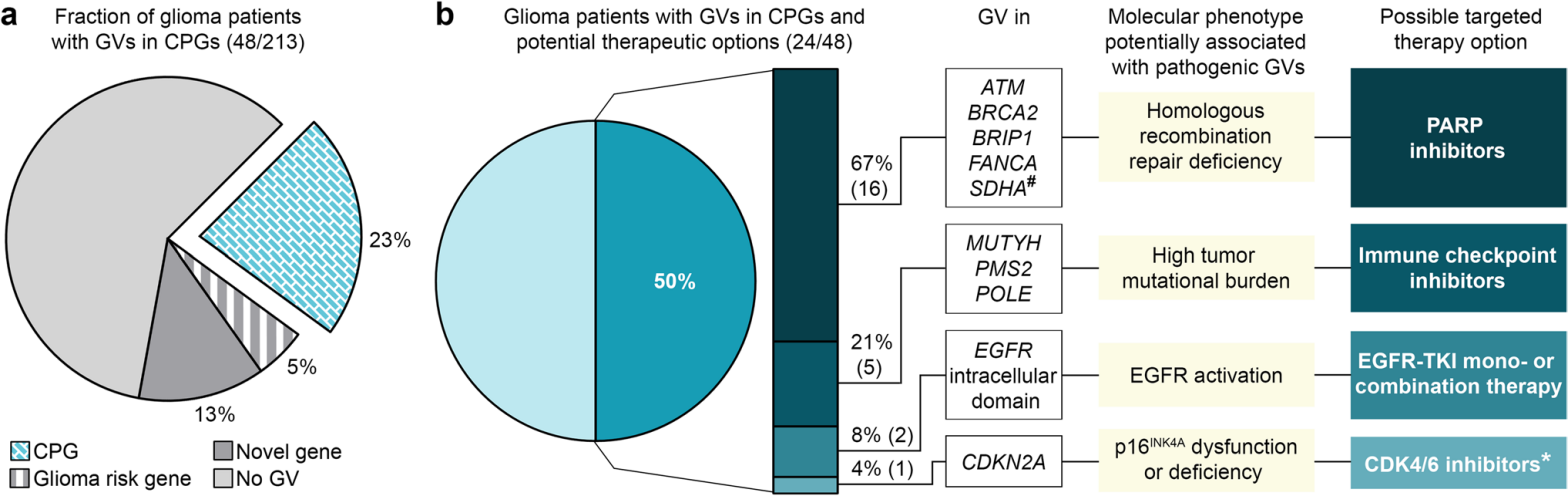
Additional treatment options may be effective in 11% (24/213) glioma patients with a familial and/or personal history of tumors. **a** The fraction of glioma patients with pathogenic GVs in CPGs is 23% (48/213). **b** For half (24/48) of the glioma patients with pathogenic GVs in CPGs, targeted treatment options exist that may potentially improve the outcome of patients with the respective GVs. ^#^, Loss-of-function variants in succinate dehydrogenase genes, including *SDHA*, lead to an accumulation of the oncometabolite succinate in the tumor that suppresses the homologous recombination DNA repair pathway [75]; ^*^, preclinical evidence only [57]; *CPG* cancer predisposition gene; *GV* germline variant; *PARP* poly(ADP-ribose) polymerase; *TKI* tyrosine kinase inhibitor

Most of the genes affected by GVs were CPGs (27/46, 58.7%, Fig. 1b, 2a). Pathogenic GVs in CPGs were detected in 48/213 (23%) glioma patients (Fig. 5a). In some tumor entities, it has been effective to target the molecular tumor phenotypes caused by 10 of the identified CPGs, if mutated, including HRR deficiency [43], high TMB [48], activation of the epidermal growth factor receptor (EGFR) [46], and p16^INK4A^ dysfunction/deficiency (preclinical evidence) [57]. As 24/48 (50%) glioma patients were affected by GVs in one of these 10 CPGs, they may be possible candidates for a molecularly targeted therapy, i.e., with PARP inhibitors (16/24, 67%), immune checkpoint inhibitors (ICI) (5/24, 21%), EGFR tyrosine kinase inhibitor (TKI) mono- or combination therapy (2/24, 8%, EGFR is expressed and Tyr1068-phosphorylated in two glioblastomas of patients with *EGFR* GVs, Supplementary Fig. 9 online resource), or CDK4/6 inhibitors (1/24, 4%) (Fig. 5b).

## Discussion

In this study, we aimed to extend the knowledge on genes and variants impacting the genetic risk for glioma development by screening 213 adult glioma patients from 206 families with presumed tumor predisposition for germline variants by WES. With respect to CPGs, our findings strongly implicate *BRCA2* pathogenic GVs in the tumorigenesis of adult gliomas (in line with [32, 51]), confirm that GVs in *ATM*, *BRIP1*, *CDKN2A*, *MUTYH*, *PMS2*, *POLE*, *SDHA*, *TP53*, and other CPGs are associated with glioma risk [15, 32, 51], and newly propose GVs in *EGFR*, *GBA1*, *GJB2*, and other CPGs as glioma predisposition candidates (Supplementary Tables 4, 5 online resource). GVs in CPGs were detected in 48 (23%) glioma patients with presumed tumor predisposition analyzed here, half of whom may be candidates for therapy options targeting the molecular tumor phenotype caused by their GVs (Fig. 5). Of the suspected glioma risk genes mutated in glioma patients here, two each were associated with cell adhesion, ion transport, vesicle transport, or immune response. Of the novel candidate genes mutated more frequently in the glioma than in the in-house control cohort, at least two had functions in metabolism or ion transport, respectively.

The *BRCA2* gene, which encodes an essential homology-directed DNA DSB repair factor [56], was one of 10 affected CPGs involved in DNA damage response, making up 37% of all mutated CPGs, and was implicated in adult glioma risk using both approaches. Heterozygous pathogenic GVs in *BRCA2* are associated with HRR deficiency and susceptibility to female breast, male breast, esophageal, gastric, pancreatic, ovarian, and prostate cancer [41, 55]. Similar to our findings, *BRCA2* variants have been linked to pediatric brain tumors. In Fanconi anemia group D1 patients with biallelic pathogenic GVs in *BRCA2*, brain tumors, particularly medulloblastoma and astrocytoma, were found in the first decade of life in almost half (15/27) of the cases [2], and glioblastoma in childhood was also reported [21, 64]. In a meta-analysis, heterozygous ClinVar LP/P *BRCA2* GVs were significantly more frequent in 876 children and adolescents with brain tumors than in a control cohort [38]. The frequency of heterozygous ClinVar pathogenic *BRCA2* GVs in unselected adult glioma patients ranged from 3/764 (0.4%) [32] to 3/152 (2%) [51], compared to 5/206 (2.4%) glioma patients with presumed tumor predisposition analyzed here. All *BRCA2* GVs identified here were LoF variants resulting in a reduced BRCA2 expression and an increased PARP1 expression, on average, in glioma sections compared to gliomas from non-*BRCA2* GV carriers, thus impacting the molecular tumor phenotype.

The most frequently mutated gene, *ATM*, encoding a serine/threonine protein kinase, is also involved in DNA DSB repair, i.e., by phosphorylating proteins to initiate DNA damage response [40]. Biallelic *ATM* GVs cause ataxia telangiectasia characterized by a predisposition to tumors, including medulloblastoma [28] and cerebellar astrocytoma [53], immunodeficiency, and cerebellar degeneration among other features [68]. Heterozygous *ATM* GV carriers also have an increased cancer risk, particularly of female breast cancer [76]. Our data of heterozygous *ATM* GVs in 2.9% glioma patients with presumed tumor predisposition corroborate and extend the findings of *ATM* GVs in 1/152 (0.7%) glioma patients [51] and 3/304 (1.0%) glioma families [15]. In this study, heterozygous GVs in *ATM* predisposed mainly to IDH-mutant astrocytoma at a median age of 36 years, compared to 58 years at primary glioma diagnosis in patients without GVs, mainly affected by glioblastoma. The significantly younger age at glioma diagnosis of *ATM* GV carriers may at least partly be explained by the younger median age of patients presenting with IDH-mutant astrocytoma (38 years) [9], compared with a peak incidence of IDH-wildtype glioblastoma between 55 and 85 years [44]. Four of 6 *ATM* GVs detected in glioma patients here were LoF variants (two were ClinVar LP/P variants) that resulted in decreased ATM expression in the glioma tissue compared to gliomas from non-*ATM* GV carriers, thus impacting the molecular phenotype of the tumor. An effect of *ATM* variants on the radio-sensitivity of gliomas was previously reported, with significantly higher 1-year in-field control rates after radiation therapy in IDH-wildtype high-grade gliomas with versus without somatic pathogenic *ATM* variants [35].

Two genes associated with the repair of DNA replication errors were also recurrently affected, e.g., *POLE*, a DNA proofreading repair gene, and *PMS2*, a mismatch repair (MMR) gene. While heterozygous GVs in *POLE* and *PMS2* cause polymerase proofreading-associated polyposis or hereditary non-polyposis colorectal cancer, respectively, predisposing to colorectal cancer [58, 59], they have also been linked to glioma risk [5, 15, 32, 34, 60, 80], in line with our data.

Around 20% of the mutated CPGs and 50% of the identified novel candidate genes of this study are functionally involved in metabolism, for instance of mitochondria (*SDHA*, *TRMT5*)*,* lysosomes (*GBA1*), and peroxisomes (*PHYH*). *SDHA* encoding a subunit of the Krebs cycle enzyme succinate dehydrogenase is among the nuclear genes associated with the Leigh syndrome spectrum [50] that impact mitochondrial energy metabolism. Heterozygous *SDHA* LoF variants cause the pheochromocytoma/paraganglioma syndrome and lead to the accumulation of the oncometabolite succinate with various cellular consequences, e.g., on HRR [39, 75, 83]. Similar effects are seen due to elevated levels of the oncometabolite D-2-hydroxyglutarate in gliomas with activating variants of the *IDH1* and *IDH2* genes encoding the isocitrate dehydrogenase enzymes [19, 39]. In this study, two LoF variants (one of which was ClinVar LP/P) in *SDHA* possibly impacting succinate levels were detected in three patients with IDH-wildtype glioblastoma suggesting that the accumulation of succinate in glioblastomas with normal D-2-hydroxyglutarate levels may have similar oncogenic effects as the accumulation of D-2-hydroxyglutarate in IDH-mutant astrocytomas, and thus contribute to glioblastoma development. Similarly, glucosylceramide and glucosylsphingosine that are stored in the CNS, among other organs, in Gaucher disease type 2 and 3 caused by biallelic GVs in the *GBA1* gene [73] may act as protumorigenic agents to increase the risk of glioblastoma and other tumors. This hypothesis would be in line with our finding of two different heterozygous ClinVar LP/P GVs in *GBA1* in three glioblastoma patients, and an increased risk of hematological malignancies and solid tumors in patients with Gaucher disease [22].

Other gene functions implicated in glioma risk in this study include signal transduction (e.g. *EGFR*), cell adhesion (e.g. *GJB2*), immune response (e.g. *IFIH1* and *SAMHD1*), and ion transport (e.g. *CFTR*). *EGFR*, encoding a transmembrane receptor tyrosine kinase acting in the PI3K/AKT/mTOR, MAPK, and other pathways, is among the genes most frequently activated by gene amplification, genetic rearrangements, and single nucleotide variants in IDH-wildtype glioblastomas [10]. While somatic *EGFR* variants in glioblastomas rarely affect the kinase domain [10], two of the three GVs in *EGFR* identified in the germline of glioma patients here and GVs in families with lung cancer affect the intracellular domain of EGFR [42]. The *GJB2* gene encodes connexin 26, a gap junction protein associated with autosomal recessive and autosomal dominant non-syndromic deafness, and autosomal dominant hearing loss syndromes combined with skin disorders [62]. Interestingly, here we detected two ClinVar LP/P *GJB2* GVs in three glioma patients, newly implicating pathogenic GVs in *GJB2* in glioma risk. If biallelically mutated, *IFIH1* and *SAMHD1* are among the genes causing the autosomal recessive Aicardi-Goutières syndrome (AGS), a type I interferonopathy leading to an early-onset progressive encephalopathy with basal ganglia calcifications [18]. While one AGS patient with biallelic *SAMHD1* GVs and chronic lymphocytic leukemia has been described [17], heterozygous GVs in *SAMHD1* have been detected in a multiple myeloma family and significantly associated with prostate cancer risk [13, 52]. Here, we identified a heterozygous ClinVar LP/P *SAMHD1* GV and a heterozygous *IFIH1* GV with a CADD score > 30 in one patient each with an IDH-mutant glioma, providing further evidence for a link between AGS genes and glioma risk that we had previously described [6]. In that report, deleterious GVs in the AGS genes *ADAR* and *RNASEH2B* were detected in families and patients with astrocytomas or glioblastomas as well as with prostate cancer [6]. Biallelic GVs in the *CFTR* gene encoding an ATP-binding cassette transporter that functions as a chloride channel cause cystic fibrosis mainly affecting the lung, gastrointestinal tract, and skin [33, 65, 67]. Cases of pancreatic cancer and adenocarcinoma of the ileum have been reported, and a susceptibility for colorectal cancer has been observed in cystic fibrosis patients [7]. In addition, the prevalence of pathogenic *CFTR* GVs in the colorectal cancer population was significantly higher than expected suggesting an increased cancer risk not only in cystic fibrosis patients, but also in heterozygous *CFTR* GV carriers [7]. Similarly, we found heterozygous *CFTR* GVs to be significantly more frequent in glioma patients with presumed tumor predisposition compared to controls, suggesting a link to glioma risk.

In this study, 24/213 (11%) glioma patients with presumed tumor predisposition carried GVs in CPGs that potentially sensitize them to targeted therapies not routinely used in glioma patients, such as PARP, immune checkpoint, EGFR, or CDK4/6 inhibitors (Fig. 5). PARP inhibitors target DNA damage repair pathways leading to synthetic lethality of tumor cells with HRR deficiency, e.g., due to variants in genes such as *ATM* or *BRCA2* [27]. Treatment with the PARP inhibitor olaparib provided a significant benefit over standard therapy in patients with a *BRCA* GV and metastatic breast or pancreatic cancer [26, 66], and a high response rate in patients with *ATM* GVs and metastatic prostate cancer [49]. Novel PARP inhibitors, such as AZD9574, are currently being developed that penetrate the blood–brain barrier and can be used in the treatment of brain tumors [72]. Blood–brain barrier penetrant PARP inhibitors may be effective in glioma patients carrying GVs in *ATM*, *BRCA2*, *BRIP1*, *FANCA*, and *SDHA* identified here that are associated with HRR deficiency, and may act synergistically with radio-, chemo-, and immunotherapy [70]. Advanced solid tumors with high TMB could have a robust response to ICI therapy [48]. Causes of high TMB include defects in MMR and polymerase proofreading repair due to pathogenic variants in *PMS2* or *POLE*, respectively [11]. Therefore, five glioma patients identified here with heterozygous GVs in genes conferring a high TMB, i.e., *PMS2*, *POLE*, and *MUTYH*, may be candidates for treatment with ICIs, such as nivolumab or pembrolizumab, shown to be effective in individual glioblastoma patients carrying homozygous *PMS2* GVs or a heterozygous *POLE* GV [8, 31], and in a pediatric trial of refractory malignancies with high TMB including glioblastoma patients with biallelic *PMS2* GVs [20]. EGFR-TKIs, such as erlotinib, have shown high response rates in patients with non-small cell lung cancer and activating variants in the intracellular EGFR kinase domain [61], and may represent a potential therapeutic option for our patients carrying *EGFR* GVs with glioblastomas expressing Tyr1068-phosphorylated EGFR, which was associated with erlotinib sensitivity in preclinical lung cancer models [69]. Interestingly, erlotinib in combination with the chemotherapeutic agent gemcitabine showed efficacy in a patient with a pancreatic ductal adenocarcinoma carrying the *EGFR*:c.2189T>G p.(L730R) GV [54] that was also detected in a glioblastoma patient here. CDK4/6 inhibitors may be a future treatment modality for glioma patients with pathogenic *CDKN2A* variants causing melanoma-astrocytoma syndrome [77] detected here in one *CDKN2A* GV carrier with an anaplastic astrocytoma and a melanoma. According to preclinical data, CDKN2A deficiency sensitizes IDH-mutant glioma to CDK4/6 inhibitors [57]. As an increasing number of compounds is under preclinical investigation for molecular targeted therapy, additional treatment options for glioma patients with GVs in other genes may arise in the future.

In conclusion, 48/213 (23%) glioma patients with a presumed tumor predisposition carried at least one deleterious heterozygous GV in a CPG, identifying a hereditary syndrome linked to an increased risk of gliomas and other tumors in them and their families with implications for surveillance and, in around half of the cases, a potential for targeted treatment options. The genes implicated in glioma risk play roles in DNA damage response, e.g., *ATM*, *BRCA2*, *PMS2*, *POLE*, or diverse other processes including metabolism, signal transduction, and cell cycle regulation. Our data provide genes of interest for germline testing in glioma patients with a familial or personal medical history of tumors.

## Supplementary Information

Below is the link to the electronic supplementary material.

Supplementary file1 (PDF 3355 KB)

## Data Availability

For reasons of confidentiality, the raw WES data of the patients cannot be shared. The remaining data generated or analyzed in this study are included in this published article and its supplementary information files.
