# Supplementary material for: Germline variants in ATM, BRCA2, other cancer predisposition and novel candidate genes are implicated in glioma risk in adult glioma patients with a familial or personal history of tumors
Source: Acta Neuropathol. 2026 Jan 17;151(1):6. doi: 10.1007/s00401-025-02972-6 (PMC12812103; doi:10.1007/s00401-025-02972-6)
Supplement: Supplementary file 1 — Supplementary file1 (PDF 3355 KB) [file 401_2025_2972_MOESM1_ESM.pdf]

Supplementary Material

Germline variants in *ATM*, *BRCA2*, other cancer predisposition and novel candidate genes are implicated in glioma risk in adult glioma patients with a familial or personal history of tumors

Frank Brand[1*], Lily S. Rose[1*], Amir H. Akbarzadeh[1], Christine A. M. Weber[1], Isabel Eckert[2,3], Gunnar Schmidt[1], Bernd Auber[1], Alisa Förster[1], Ulrike Beyer[1], Robert Geffers[4], Stephan Bartels[5], Michael Lalk[6], Manolis Polemikos[3], Michael Friese[7], Michael Sabel[8,9], Philipp Schwenkenbecher[10], Paul Kremer[11], Arya Nabavi[6], Amir Samii[12], Ulrich Lehmann[5], Guido Reifenberger[13,14], Joachim K. Krauss[3], Bettina Wiese[2,3], Christian Hartmann[15], Ruthild G. Weber[1]

[1]Department of Human Genetics, Hannover Medical School, Hannover, Germany
[2]Department of Neurology, Henriettenstift, Diakovere Krankenhaus gGmbH, Hannover, Germany
[3]Department of Neurosurgery, Hannover Medical School, Hannover, Germany
[4]Genome Analytics Research Group, Helmholtz Centre for Infection Research, Braunschweig, Germany
[5]Institute of Pathology, Hannover Medical School, Hannover, Germany
[6]Department of Neurosurgery, KRH Klinikum Nordstadt, Hannover, Germany
[7]Department of Pathology and Neuropathology, Asklepios Klinik Nord - Heidberg, Hamburg, Germany
[8]Department of Neurosurgery, University Hospital Düsseldorf and Heinrich Heine University, Medical Faculty, Düsseldorf, Germany
[9]Beta Klinik, Bonn, Germany
[10]Department of Neurology, Hannover Medical School, Hannover, Germany
[11]Department of Neurosurgery, Asklepios Klinik Nord - Heidberg, Hamburg, Germany
[12]Department of Neurosurgery, International Neuroscience Institute, Hannover, Germany
[13]Institute of Neuropathology, University Hospital Düsseldorf and Heinrich Heine University, Medical Faculty, Düsseldorf, Germany
[14]German Cancer Consortium (DKTK), Partner Site Essen/Düsseldorf and German Cancer Research Center (DKFZ), Heidelberg, Germany
[15]Department of Neuropathology, Institute of Pathology, Hannover Medical School, Hannover, Germany

Running title: *ATM*, *BRCA2*, and novel genes in adult glioma risk

Correspondence to: Ruthild G. Weber, M.D., Department of Human Genetics OE 6300, Hannover Medical School, Carl-Neuberg-Str. 1, 30625 Hannover, Germany, Phone +49 511 532 7751, Fax +49 511 532 18520, Email: Weber.Ruthild@mh-hannover.de

*These authors contributed equally as first authors

Supplementary Tables

Supplementary Table 1. List of genes analyzed in approach 1

**Cancer predisposition genes (n=114)***
ABCB11, ALK, APC, ATM, AXIN2, BAP1, BLM, BMPR1A, BRCA1, BRCA2, BRIP1, BUB1B, CBL, CDC73, CDH1, CDK4, CDKN1B, CDKN2A, CEBPA, CHEK2, COL7A1, CYLD, DDB2, DICER1, DIS3L2, DKC1, DOCK8, EGFR, ELANE, ERCC2, ERCC3, ERCC4, ERCC5, EXT1, EXT2, FAH, FANCA, FANCC, FANCG, FH, FLCN, GATA2, GBA1, GJB2, GPC3, HFE, HMBS, HRAS, ITK, KIT, MAX, MEN1, MET, MLH1, MSH2, MSH6, MTAP, MUTYH, NBN, NF1, NF2, PALB2, PDGFRA, PHOX2B, PMS2, POLD1, POLE, POLH, PRKAR1A, PRSS1, PTCH1, PTEN, PTPN11, RAD51C, RAD51D, RB1, RECQL4, RET, RHBDF2, RMRP, RUNX1, SBDS, SDHA, SDHAF2, SDHB, SDHC, SDHD, SERPINA1, SH2D1A, SLC25A13, SMAD4, SMARCA4, SMARCB1, SMARCE1, SOS1, SRY, STAT3, STK11, SUFU, TERT, TGFBR1, TMEM127, TNFRSF6, TP53, TRIM37, TSC1, TSC2, UROD, VHL, WAS, WRN, WT1, XPA, XPC
**Glioma risk genes (n=50)#**
ADAR, ADAMTS8, BAP1, BARD1, CASC4, CASC5, CCDC26, CMTF1, COL4A1, CTNND1, DCAF8L2, DDN, DMBT1, EPCAM, FLOT2, FOCAD, GREM1, HERC2, HP1BP3, IFIH1, IGSF9, IP6K1, KAT5, KIAA1549, LRRK2, LSM11, MAST4, MYO7A, PC, PCNXL4, PCYT1A, PIK3R4, POT1, PPP1R16B, RNASEH2A, RNASEH2B, RNASEH2C, RNU7-1, SAMHD1, SCG5, SESTD1, SLC4A7, TFAP2E, THSD7A, TREX1, TRPC4AP, TRPM1, WDR7, XRCC2, ZC3H7B

*Based on Rahman 2014; additional references: Förster et al. 2021; Weber et al. 2023
#Based on Bainbridge et al. 2015; Beyer et al. 2017; Brand et al. 2020; Brockschmidt et al. 2012; Catalano et al. 2021; Choi et al. 2023; Crow and Stetson 2022; Mitchell et al. 2025

Supplementary Table 2a. Clinical features and tumor characteristics of 56 glioma patients from 54 families carrying at least one pathogenic germline variant in a cancer predisposition gene and/or a glioma risk gene, and tumors diagnosed in family members: results from approach 1

Patient ID	Gene	Germline variant	Sex	Primary glioma					Non-brain tumors diagnosed in the patient	Tumors diagnosed in family members
				Age at Dx[a]	Localization	Histology	WHO grade[b]	Molecular characteristics		
WI99-III.1	*APC*	p.(P1960L)	F	60	Insula (L)	Glioblastoma	IV	IDH-WT	-	Breast tumor
WI89-III.1	*APC*<br>*SLC4A7*	p.(G2164V)<br>p.(Y960C)	F	46	Pons	Anaplastic astrocytoma[c]	III	NA	Thyroid tumor	Brain tumor NOS, colon cancer
WI105-III.1	*ATM*	c.901+2T>A	M	48	Thalamus (L)	Diffuse midline glioma[c]	IV	IDH-WT H3 K27M-mut	-	Prostate cancer
WI37-III.1	*ATM*	p.(Y1957C)	M	25	Cerebellopontine angle	Astrocytoma	3	IDH-mut	-	Thyroid tumor, neck tumor
Fam004-III.1	*ATM*	p.(A2274T)	M	65	Frontal (L)	Glioblastoma	IV	IDH-WT	-	Glioblastoma
WI207-III.1	*ATM*	c.7630-2A>C	M	35	Parietal (R)	Astrocytoma	2	IDH-mut	-	Meningioma
WI166-III.1	*ATM*<br>*GBA1*	p.(D2625_A2626delinsEP)<br>p.(N409S)	F	22	Frontal (R)	Astrocytoma	4	IDH-mut	-	Stomach tumor
WI160-III.1	*ATM*	p.(L3010*)	F	37	Temporoparietal (R)	Anaplastic astrocytoma	III	IDH-mut	-	Breast tumor, uterine tumor
WI14-III.1	*BRCA2*	c.316+5G>C	M	30	Precentral (L)	Anaplastic astrocytoma	III	IDH-mut	-	Breast cancer, small-cell lung cancer
WI60-III.1	*BRCA2*	p.(Q742*)	M	49	Temporal (R)	Astrocytoma	4	IDH-mut	-	Colon tumor
WI226-III.1	*BRCA2*	p.(K944*)	F	63	Parietal	Glioblastoma	4	IDH-WT	Melanoma	Basal cell carcinoma, breast cancer, lung tumor
WI175-III.1	*BRCA2*	p.(I1470Kfs*11)	M	59	Frontal (R)	Glioblastoma	IV	IDH-WT	Basal cell carcinoma	Bladder cancer, breast cancer
WI191-III.1	*BRCA2*<br>*COL7A1*	p.(S3366Nfs*4)<br>p.(V2448V)	F	65	Frontal (R)	Anaplastic oligodendroglioma	III	IDH-mut, 1p/19q-codel	-	Brain tumor NOS
WI86-III.1	*BRIP1*	c.205+5G>T	M	29	Frontal (L)	Anaplastic oligodendroglioma	III	IDH-mut, 1p/19q-codel	-	Colon cancer

Fam002-III.1			M	37	Frontal (L)	Oligodendroglioma	II	IDH-mut, 1p/19q-codel	-	
Fam002-III.2	*CDH1*	p.(A817V)	F	38	Frontal (L)	Anaplastic oligodendroglioma	III	IDH-mut, 1p/19q-codel	-	Kidney tumor
Fam002-II.2			M	51	Frontal (L)	Oligodendroglioma	II	IDH-mut, 1p/19q-codel	-	
WI04-III.1	*CDKN2A*	p.(M53I)	F	72	Precentral (L)	Anaplastic astrocytoma[c]	III	NA	Melanoma	-
WI122-III.1	*CTNND1*	p.(R885W)	F	26	IV ventricle	Pilocytic astrocytoma[c]	I	NA	Lymphoma	-
WI61-III.1	*DICER1*	p.(I846S)	F	72	Frontal (L)	Anaplastic astrocytoma[c]	III	IDH-WT	Breast cancer	-
WI239-III.1	*DICER1*	p.(Y1835C)	F	68	Parietal (R)	Glioblastoma	4	IDH-WT	-	Glioma NOS
WI163-III.1	*DIS3L2*	p.(R274Q)	M	85	Parietooccipital (L)	Glioblastoma	IV	IDH-WT	-	Abdominal tumor, adrenal tumor, brain tumor NOS
WI201-III.1	*EGFR*	p.(C251S)	M	58	Temporal (R)	Glioblastoma	4	IDH-WT	-	Bladder tumor, prostate cancer
WI177-II.1	*EGFR*	p.(L730R)	M	61	Parietal	Glioblastoma	IV	IDH-WT	Testicular cancer	Anaplastic astrocytoma, breast cancer
WI33-III.1	*EGFR*	p.(R962H)	F	22	Frontal (R)	Astrocytoma	II	IDH-mut	-	Breast cancer
WI165-III.1	*EPCAM*	p.(R138*)	M	82	Frontal (R)	Glioblastoma	IV	IDH-WT	Bladder tumor, melanoma	-
WI153-III.1	*ERCC2*	p.(D681N)	M	45	Temporoparietal (L)	Glioblastoma	IV	IDH-WT	-	Breast cancer, colon cancer
WI126-II.2	*ERCC3*	p.(I407S)	F	57	Frontal (L)	Glioblastoma	IV	IDH-WT	-	Colon tumor, leukemia
WI72-III.1	*ERCC5*	p.(R138*)	M	49	Frontal (R)	Oligodendroglioma	II	IDH-mut, 1p/19q-codel	Prostate cancer	-
WI50-III.1	*FAH*	c.1062+5G>A	M	58	Parietal (R)	Oligodendroglioma	II	IDH-mut, 1p/19q-codel	-	Colon cancer
WI209-III.1	*FANCA*	p.(T1131A)	M	73	Frontal (R)	Glioblastoma	4	IDH-WT	-	Brain tumor NOS, prostate tumor, testicular tumor

WI22-III.2	*FLCN*	p.(S406G)	F	47	Parietal (L)	Glioblastoma	IV	IDH-WT	-	Basal cell carcinoma, melanoma, oral squamous cell carcinoma
	PCYT1A	p.(R335*)								
WI205-III.1	*GBA1*	p.(N409S)	M	73	Bi-hemispheric	Glioblastoma	4	IDH-WT	-	Breast tumor, esophageal tumor, stomach tumor
WI214-III.1	*GBA1*	p.(N409S)	M	61	Parietooccipital (L)	Glioblastoma	4	IDH-WT	-	Brain tumor NOS, colon cancer, laryngeal tumor, tumor NOS
WI09-III.1	*GBA1*	p.(R502H)	F	73	Frontal (R)	Glioblastoma	IV	IDH-WT	Neuroendocrine tumor in the digestive tract	-
WI48-III.1	*GJB2*	p.(V37I)	F	28	Frontal (R)	Astrocytoma	II	IDH-mut	-	Leukemia
	TRPM1	p.(D1392Lfs*11)								
WI49-III.1	*GJB2*	p.(V37I)	M	52	Frontal (R)	Glioblastoma[c]	IV	NA	-	Brain tumor NOS
WI75-III.1	*GJB2*	p.(Y155*)	M	53	Temporooccipital (L)	Glioblastoma	IV	IDH-WT	-	Breast cancer
WI169-II.2	*HMBS*	p.(R32H)	F	55	Frontal (L)	Glioblastoma	IV	IDH-WT	Eye tumor	Brain tumor NOS
WI106-III.1	*IFIH1*	p.(R598C)	M	38	Frontal	Oligodendroglioma	II	IDH-mut, 1p/19q-codel	-	Breast cancer
WI53-III.1	*KAT5*	p.(R448*)	M	61	Frontal (L)	Anaplastic oligodendroglioma	III	IDH-mut, 1p/19q-codel	-	Colon cancer
WI70-III.1	*MUTYH*	p.(R217H)	M	75	Frontoparietal (R)	Glioblastoma	IV	IDH-WT	-	Brain tumor NOS, small-cell lung cancer
Fam003-III.1	*MYO7A*	p.(G1159V)	M	79	Frontal (R)	Glioblastoma	IV	IDH-WT	Testicular tumor (benign)	Glioblastoma
WI222-III.1	*NF2*	p.(R588*)	F	72	Frontal (L)	Glioblastoma	4	IDH-WT	Breast cancer, ovarian & uterine tumors (benign)	Brain tumor NOS, breast tumor, skin cancer
WI103-III.1	*PMS2*	p.(I292F)	F	39	Central (L)	Oligodendroglioma[c]	III	IDH-mut, NA	Skin tumor	-
WI11-III.1	*PMS2*	p.(V306A)	F	72	Frontal (R)	Glioblastoma	IV	IDH-WT	Breast cancer	Bladder cancer, stomach tumor

WI88-III.1	*POLE*	p.(M1L)	M	55	Frontal (L)	Astrocytoma	II	IDH-mut	-	Breast tumor, stomach tumor
Fam016-III.1	*POLE*	p.(R1136W)	M	44	Temporooccipital (L)	Glioblastoma	IV	IDH-WT	-	Breast cancer, glioblastoma
WI78-III.1	*SAMHD1*	p.(S23*)	F	35	Cerebellopontine angle (L)	Astrocytoma	2	IDH-mut	-	Hodgkin lymphoma
WI87-III.1	*SDHA*	p.(R31*)	F	67	Frontal (L)	Glioblastoma	IV	IDH-WT	-	Kidney cancer, tumor NOS
LI06-III.1	*SDHA*<br>*GATA2*	p.(R31*)<br>p.(W10C)	F	70	Parietal (L)	Glioblastoma	IV	IDH-WT	-	Colon tumor, skin cancer
WI183-III.1	*SDHA*	c.1795-3C>G	M	64	Temporal (R)	Glioblastoma	IV	IDH-WT	-	Stomach tumor
WI145-III.1	*SLC25A13*	p.(G283E)	M	40	Temporal (L)	Astrocytoma	II	IDH-mut	-	Testicular cancer, tumor NOS
WI51-III.1	*TFAP2E*	p.(S298L)	M	66	Bi-hemispheric	Glioblastoma	IV	IDH-WT	Basalioma	-
WI236-III.1	*TP53*	p.(N131S)	F	46	Operculum (R)	Glioblastoma	4	IDH-WT	Breast tumor	Breast cancer
WI08-III.1	*WDR7*	p.(P1064L)	M	61	Parietooccipital (R)	Glioblastoma	IV	IDH-WT	-	Colon cancer, stomach cancer

Codel, codeleted; Dx, diagnosis; F, female; fs, frameshift; IDH, isocitrate dehydrogenase 1/2; L, left; M, male; mut, mutant; NA, not available; NOS, not otherwise specified; R, right; WHO, World Health Organization; WT, wildtype; *, stop codon

[a]Age at diagnosis of primary glioma in years

[b]WHO grade of primary glioma according to the WHO classification used at the time: roman numerals are used in the WHO classification of 2007 and 2016, arabic numerals are used in the WHO classification of 2021

[c]This glioma was grouped into the category “other glioma” because it could not be classified as a “glioblastoma, IDH-wildtype”, an “astrocytoma, IDH-mutant”, or an “oligodendroglioma, IDH-mutant and 1p/19q-codeleted”

Supplementary Table 2b. Additional tumor characteristics and survival data of 56 glioma patients from 54 families carrying at least one pathogenic germline variant in a cancer predisposition gene and/or a glioma risk gene determined by approach 1

Case	Germline variant(s)	Histology of the primary glioma	Histology of the recurrent glioma	Molecular characteristics of primary (p) or recurrent (r) glioma including second hit and tumor mutational burden (TMB)	Survival (mo)	
					PFS	OS
WI99-III.1	*APC* p.(P1960L)	Glioblastoma	NA	- IDH-WT (p)<br>- *CDKN2A/B* deletion (p)<br>- *EGFR* amplification and point mutation (extracellular domain) (p)	NA	NA
WI89-III.1	*APC* p.(G2164V), *SLC4A7* p.(Y960C)	Anaplastic astrocytoma[a]	NA	NA	NA	165
WI105-III.1	*ATM* c.901+2T>A	Diffuse midline glioma[a]	NA	- IDH-WT (p)<br>- Loss of nuclear ATRX expression (p)<br>- H3 K27-altered (*H3F3A* p.(K27M)) (p)	NA	14
WI37-III.1	*ATM* p.(Y1957C)	Astrocytoma	NA	- IDH-mut (*IDH1* p.(R132G)) (p)<br>- Loss of nuclear ATRX expression (p)	NA	NA
Fam004-III.1	*ATM* p.(A2274T)	Glioblastoma	NA	- IDH-WT (p)	NA	11
WI207-III.1	*ATM* c.7630-2A>C	Astrocytoma	NA	- IDH-mut (*IDH1* p.(R132H)) (p)<br>- Loss of nuclear ATRX expression (p)<br>- Strong nuclear expression of p53 (p)	NA	NA
WI166-III.1	*ATM* p.(D2625_A2626delinsEP), *GBA1* p.(N409S)	Astrocytoma	NA	- IDH-mut (*IDH1* p.(R132H)) (p)<br>- Loss of nuclear ATRX expression (p)<br>- *TP53* p.(F134V) (p)<br>- *MGMT* promoter methylation (p)	NA	NA
WI160-III.1	*ATM* p.(L3010*)	Anaplastic astrocytoma	Astrocytoma	- IDH-mut (p, r)<br>- Loss of nuclear ATRX expression (r)<br>- Strong nuclear expression of p53 (r)<br>- Homozygous *CDKN2A* deletion (r)	51	53
WI14-III.1	*BRCA2* c.316+5G>C	Anaplastic astrocytoma	NA	- IDH-mut (*IDH1* p.(R132C)) (p)<br>- Strong nuclear expression of p53 (r)<br>- EGFR overexpression (r)<br>- Second hit in *BRCA2*: - (p)	67	NA

WI60-III.1	*BRCA2* p.(Q742*)	Astrocytoma	NA	-IDH-mut (*IDH1* p.(R132H)) (p)<br>- Loss of nuclear ATRX expression (p)<br>- Strong nuclear expression of p53 (p)<br>- Second hit in *BRCA2*: - (p)	NA	NA
WI226-III.1	*BRCA2* p.(K944*)	Glioblastoma	NA	- IDH-WT (p)<br>- Second hit in *BRCA2*: - (p)	NA	NA
WI175-III.1	*BRCA2* p.(I1470Kfs*11)	Glioblastoma	NA	- IDH-WT (p)	NA	13
WI191-III.1	*BRCA2* p.(S3366Nfs*4), *COL7A1* p.(V2448V)	Anaplastic oligodendroglioma	Anaplastic oligodendroglioma	- IDH-mut, 1p/19q-codel (p)<br>- *MGMT* promoter methylation (p)<br>- Strong nuclear expression of p53 (p, r)<br>- Reduced nuclear expression of H3 p.K27me3 (p, r)<br>- Second hit in *BRCA2*: - (p)	13	NA
WI86-III.1	*BRIP1* c.205+5G>T	Anaplastic oligodendroglioma	Anaplastic oligodendroglioma	- IDH-mut (*IDH1* p.(R132H)), 1p/19q-codel (p, r)	34	76
Fam002-III.1	*CDH1* p.(A817V)	Oligodendroglioma	Oligodendroglioma	- IDH-mut, 1p/19q-codel (p, r)	121	NA
Fam002-III.2	*CDH1* p.(A817V)	Anaplastic Oligodendroglioma	NA	- IDH-mut, 1p/19q-codel (p)	NA	NA
Fam002-II.2	*CDH1* p.(A817V)	Oligodendroglioma	Anaplastic Oligodendroglioma	- IDH-mut, 1p/19q-codel (p, r)	24	NA
WI04-III.1	*CDKN2A* p.(M53I)	Anaplastic astrocytoma[a]	NA	NA	NA	NA
WI122-III.1	*CTNND1* p.(R885W)	Pilocytic astrocytoma[a]	NA	NA	NA	NA
WI61-III.1	*DICER1* p.(I846S)	Anaplastic astrocytoma[a]	NA	- IDH-WT (p)	NA	3
WI239-III.1	*DICER1* p.(Y1835C)	Glioblastoma	NA	- IDH-WT (p)	NA	NA
WI163-III.1	*DIS3L2* p.(R274Q)	Glioblastoma	NA	- IDH-WT (p)	NA	5
WI201-III.1	*EGFR* p.(C251S)	Glioblastoma	NA	- IDH-WT (p)<br>- *TERT* promoter mutation (*TERT*: p.(C228T)) (p)<br>- H3 K27-altered (p)<br>- *EGFR* amplification, suspected (p)	NA	19

WI177-II.1	*EGFR* p.(L730R)	Glioblastoma	NA	- IDH-WT (p)<br>- *MGMT* promoter methylation (p)<br>- *TERT* promoter mutation (*TERT* p.(C228T)) (p)	NA	10
WI33-III.1	*EGFR* p.(R962H)	Astrocytoma	Anaplastic astrocytoma	- IDH-mut (*IDH1* p.(R132G)) (p, r)<br>- Loss of nuclear ATRX expression (r)<br>- Strong nuclear expression of p53 (r)	23	48
WI165-III.1	*EPCAM* p.(R138*)	Glioblastoma	NA	- IDH-WT (p)	NA	13
WI153-III.1	*ERCC2* p.(D681N)	Glioblastoma	NA	- IDH-WT (p)<br>- *MGMT* promoter methylation (p)	NA	NA
WI126-II.2	*ERCC3* p.(I407S)	Glioblastoma	NA	- IDH-WT (p)<br>- *MGMT* promoter methylation (p)	NA	19
WI72-III.1	*ERCC5* p.(R138*)	Oligodendroglioma	Oligodendroglioma	- IDH-mut, 1p/19q-codel (p, r)<br>- *MGMT* promoter methylation (r)<br>- Heterozygous *CDKN2A* deletion (r)<br>- Loss of p16 expression in most tumor cells (r)<br>- Reduced nuclear expression of H3 p.K27me3 (r)	40	NA
WI50-III.1	*FAH* c.1062+5G>A	Oligodendroglioma	NA	- IDH-mut (*IDH1* p.(R132H)), 1p/19q-codel (p)	NA	NA
WI209-III.1	*FANCA* p.(T1131A)	Glioblastoma	NA	- IDH-WT (p)	NA	5
WI22-III.2	*FLCN* p.(S406G),<br>*PCYT1A* p.(R335*)	Glioblastoma	Glioblastoma	- IDH-WT (p, r)<br>- *MGMT* promoter methylation (p)<br>- +7/-10 chromosome copy-number alteration (p)<br>- *PTEN* deletion (p)	6	21
WI205-III.1	*GBA1* p.(N409S)	Glioblastoma	NA	- IDH-WT (p)<br>- *MGMT* promoter methylation (p)	NA	NA
WI214-III.1	*GBA1* p.(N409S)	Glioblastoma	NA	- IDH-WT (p)<br>- Strong nuclear expression of p53 (p)	NA	6
WI09-III.1	*GBA1* p.(R502H)	Glioblastoma	NA	- IDH-WT (p)<br>- *MGMT* promoter methylation (p)	NA	NA

WI48-III.1	*GJB2* p.(V37I), *TRPM1* p.(D1392Lfs*11)	Astrocytoma	Astrocytoma	- IDH-mut (*IDH1* p.(R132H)) (p, r)<br>- Loss of nuclear ATRX expression (r)<br>- (Partial) strong nuclear expression of p53 (p, r)	120	NA
WI49-III.1	*GJB2* p.(V37I)	Glioblastoma[a]	NA	NA	NA	NA
WI75-III.1	*GJB2* p.(Y155*)	Glioblastoma	NA	- IDH-WT (p)<br>- *MGMT* promoter methylation (p)	NA	NA
WI169-II.2	*HMBS* p.(R32H)	Glioblastoma	NA	- IDH-WT (p)<br>- *MGMT* promoter methylation (p)<br>- Individual multinucleated giant cells (p)	NA	NA
WI106-III.1	*IFIH1* p.(R598C)	Oligodendroglioma	Oligodendroglioma	- IDH-mut (*IDH1* p.(R132H)), 1p/19q-codel (p, r)	32	NA
WI53-III.1	*KAT5* p.(R448*)	Anaplastic oligodendroglioma	NA	- IDH-mut (*IDH1* p.(R132H)), 1p/19q-codel (p)	NA	NA
WI70-III.1	*MUTYH* p.(R217H)	Glioblastoma	NA	- IDH-WT (p)<br>- *TP53* p.(K139N) (p)<br>- *MGMT* promoter methylation (p)<br>- Second hit in *MUTYH*: - (p)<br>- TMB: 8.59 mut/Mb (p)	NA	14
Fam003-III.1	*MYO7A* p.(G1159V)	Glioblastoma	Glioblastoma	- IDH-WT (p)<br>- Strong nuclear expression of p53 (p)	2	NA
WI222-III.1	*NF2* p.(R588*)	Glioblastoma	NA	- IDH-WT (p)	NA	7
WI103-III.1	*PMS2* p.(I292F)	Oligodendroglioma[a]	Oligodendroglioma	- IDH-mut (p)	91	106
WI11-III.1	*PMS2* p.(V306A)	Glioblastoma	NA	- IDH-WT (p)<br>- *TP53* p.(H179L) (p)<br>- *DPYD* p.(M166V), p.(S534N) (p)<br>- EGFR overexpression (p)<br>- TMB: 6.11 mut/Mb (p)	NA	3
WI88-III.1	*POLE* p.(M1L)	Astrocytoma	Astrocytoma, NA	- IDH-mut (*IDH1* p.(R132H)) (p)<br>- *TP53* p.(Y220C) (p)<br>- *MGMT* promoter methylation (r)<br>- *CDKN2A* deletion (r)<br>- Loss of nuclear ATRX expression (r)<br>- (Partial) strong nuclear expression of p53 (r)<br>- TMB: 8.45 mut/Mb (p)	34	NA

Fam016-III.1	*POLE* p.(R1136W)	Glioblastoma	NA	- IDH-WT (p)<br>- *MDM2* amplification, suspected (p)<br>- *AKT1* amplification, suspected (p)<br>- TMB: 6.02 mut/Mb (p)	NA	15
WI78-III.1	*SAMHD1* p.(S23*)	Astrocytoma	NA	- IDH-mut (*IDH1* p.(R132C), p.(I130V)) (p)<br>- *TP53* p.(Y220C), p.(Y220H) (p)<br>- Strong nuclear expression of p53 (p)	NA	NA
WI87-III.1	*SDHA* p.(R31*)	Glioblastoma	Gliosarcoma	- IDH-WT (p, r)	NA	NA
LI06-III.1	*SDHA* p.(R31*), *GATA2* p.(W10C)	Glioblastoma	Glioblastoma	- IDH-WT (p)	NA	NA
WI183-III.1	*SDHA* c.1795-3C>G	Glioblastoma	Glioblastoma	- IDH-WT (p)	NA	NA
WI145-III.1	*SLC25A13* p.(G283E)	Astrocytoma	Astrocytoma, NA	- IDH-mut (p)	7	77
WI51-III.1	*TFAP2E* p.(S298L)	Glioblastoma	NA	- IDH-WT (p)<br>- (Partial) strong nuclear expression of p53 (p)	NA	5
WI236-III.1	*TP53* p.(N131S)	Glioblastoma	NA	- IDH-WT (p)<br>- *MGMT* promoter methylation (p)<br>- *TERT* promoter mutation (*TERT* p.(C228T)) (p)<br>- Second hit in *TP53*: *TP53* p.(Q104*) (p)	NA	NA
WI08-III.1	*WDR7* p.(P1064L)	Glioblastoma	Glioblastoma	- IDH-WT (p, r)	22	NA

Codel, codeleted; IDH, isocitrate dehydrogenase 1/2; mut, mutant; NA, not available; OS, overall survival; PFS, progression-free survival; TMB, tumor mutational burden; WT, wildtype; *, stop codon

[a]This glioma was grouped into the category “other glioma” because it could not be classified as a “glioblastoma, IDH-wildtype”, an “astrocytoma, IDH-mutant”, or an “oligodendroglioma, IDH-mutant and 1p/19q-codeleted”

Supplementary Table 3. Genes significantly enriched for LoF GVs and non-silent GVs with a CADD score ≥30 that are ultrarare (MAF <0.0001) or LP/P according to the ClinVar database (referred to as pathogenic GVs) in the glioma compared to the control cohort: results from approach 2

Gene	No. of pathogenic GVs in		Comparison of no. of pathogenic GVs in glioma vs. control cohort[a]	Pathogenic GVs in glioma cohort[b]	Family ID (glioma cohort)
	glioma cohort (n=206 families)	control cohort (n=391 families)			
BRCA2	5	0	0.0233	c.316+5G>C[c], p.(Q742*)[c], p.(K944*)[c], p.(I1470Kfs*11)[c], p.(S3366Nfs*4)	WI14[d], WI60[d], WI226[d], WI175[d], WI191[d]
CFTR	5	0	0.0233	p.(Y109*)[c], c.489+3A>G, p.(G542*)[c], p.(R1158*)[c], p.(Q1476*)[c]	WI192, WI21, WI58, WI169[e], WI14[e]
PHYH	4	0	0.0344	c.135-2A>G (n=2)[c], p.(D177G), p.(G227R)	LI07, WI54, WI103[e], WI106[f,g]
TRMT5	4	0	0.0344	p.(I105Sfs*4) (n=4)	WI110, WI117, WI121, WI175[e]
MAP3K20	3	0	0.0448	p.(C346*), p.(G355V), p.(S568C)	Fam012, WI45, WI105[e]
MYO1G	3	0	0.0448	p.(R155C), p.(Y624*), p.(R880C)	WI153[e], WI06, WI22[e,f]
SERPINA3	3	0	0.0448	p.(F221Pfs*7) (n=2), p.(V378Sfs*9)	WI04[e], WI32, WI71
TG	3	0	0.0448	p.(R1530*)[c], p.(C2154*), p.(A341Vfs*21)[c]	WI106[f,g], WI19, WI62
TMC1	3	0	0.0448	c.1763+3A>G (n=3)[c]	Fam008, LI06[e], WI215

CADD score, combined annotation dependent depletion score; GV, germline variant; LoF, loss-of-function; LP, likely pathogenic; MAF, minor allele frequency; no., number; P, pathogenic

[a]Listed are *Q* values obtained by a false discovery rate (10%) approach according to Benjamini, Krieger, and Yekutieli based on *P* values calculated by Fisher's exact test

[b]LoF variants included start-loss, frameshift, splice region (±5) with an effect on splicing according to MaxEntScan, and stop-gain variants, but excluded in-frame indels; a CADD (https://cadd.gs.washington.edu) score ≥30 is equivalent to the top 0.1% most deleterious variants; MAF values were retrieved from the Genome Aggregation Database (gnomAD) browser v4.1.0, non-Finnish European population (https://gnomad.broadinstitute.org)

[c]Classified as LP/P according to the ClinVar database (https://www.ncbi.nlm.nih.gov/clinvar)

[d]This variant was also identified in approach 1 (Table 1, Supplementary Table 2a, b)

[e]The index patient also carries a pathogenic GV in a cancer predisposition gene (Table 1, Supplementary Table 2a, b)

[f]The index patient also carries a pathogenic GV in a glioma risk gene (Table 1, Supplementary Table 2a, b)

[g]Index patient with pathogenic GVs in *PHYH* and *TG*

Supplementary Table 4. Comparison of our study and other reports with respect to established cancer predisposition / associated genes mutated in the germline of (familial) glioma patients

	**This study**	**Jonsson et al. 2019**	**Choi et al. 2023**	**McDonald et al. 2023**
Number and type of studied cases	n=206<br>families with ≥1 glioma patient: glioma families, brain tumor families, tumor families, glioma patients with multiple tumors (≥1 syn- or metachronous non-brain tumor) but an unremarkable family history	n=764 (cases with germline analysis)<br>glioma patients (family history of tumors not documented)	n=304<br>glioma families	n=152<br>glioma patients (some with family history of tumors)
Number of established CPGs / cancer-associated genes considered	n=114<br>Known CPGs[a]	n=341-468[b]<br>Cancer-associated genes	n=32<br>Known CPGs	n=46<br>Known CPGs
Number of families with GVs in CPGs / cancer-associated genes designated as pathogenic	At least one pathogenic GV in 46/206 (22.3%) families	At least one deleterious or likely deleterious GV in 103/764 (13.5%) families	LoF or previously identified likely pathogenic or pathogenic GVs in 15/304 (4.9%) families	Pathogenic GVs in 15/152<br>(9.8%) families
Mutated CPGs / cancer-associated genes[c]	***APC***, ***ATM***, ***BRCA2***, ***BRIP1***, *CDH1*, ***CDKN2A***, *COL7A1*, *DICER1*, *DIS3L2*, *EGFR*, ***ERCC2***, *ERCC3*, ***ERCC5***, *FAH*, *FANCA*, ***FLCN***, *GATA2*, *GBA1*, *GJB2*, *HMBS*, ***MUTYH***, *NF2*, ***PMS2***, ***POLE***, ***SDHA***, *SLC25A13*, ***TP53***	*ALOX12B*, ***APC***, *AR*, *ATR*, *BARD1*, *BLM*, ***BRCA2***, ***BRIP1***, *CASP8*, *CD274*, *CHEK1*, ***CHEK2***, *CSF3R*, *EPCAM*, ***ERCC2***, ***ERCC5***, *FAM175A*, *FANCC*, *FAT1*, *FGFR1*, *FGFR3*, *FH*, ***FLCN***, *FOXP1*, *HIST1H3B*, *HLA-A*, *IL7R*, *LATS2*, *MITF*, *MPL*, *MRE11A*, ***MSH2***, *MSH6*, ***MUTYH***, ***NBN***, ***NF1***, *NOTCH3*, *NOTCH4*, *PALB2*, *PARK2*, *PIK3C2G*, *PMS1*, ***PMS2***, *PTCH1*, *PTEN*, *PTPN11*, *RAD50*, *RAD51*, *RB1*, ***SDHA***, *SDHAF2*, *SH2B3*, *SMAD3*, *SOX17*, *TCF3*, *TGFBR2*, *TMEM127*, ***TP53***, *XRCC2*	***ATM***, ***BRCA1***, ***BRIP1***, ***CDKN2A***, *MLH1*, ***NBN***, ***PMS2***, *POLD1*, ***POLE***	***ATM***, ***BRCA1***, ***BRCA2***, ***CHEK2***, ***MSH2***, *MSH3*, ***MUTYH***, ***NF1***

CPG, cancer predisposition gene; GV, germline variant; LoF, loss-of-function
[a]Listed in Supplementary Table 1
[b]Three versions of the MSK-IMPACT sequencing platform were used, targeting 341, 410, or 468 genes, respectively
[c]Genes affected by deleterious GVs in more than one study are given in bold print and listed in Supplementary Table 5

Supplementary Table 5. Cancer predisposition / associated genes mutated in the germline of (familial) glioma patients in two or three of the four studies listed in Supplementary Table 4

Gene	Number of studies with germline variants in (familial) glioma patients[a]
ATM	**3**
BRCA2	**3**
BRIP1	**3**
MUTYH	**3**
PMS2	**3**
APC	2
BRCA1	2
CDKN2A	2
CHEK2	2
ERCC2	2
ERCC5	2
FLCN	2
NF1	2
POLE	2
SDHA	2
TP53	2

[a]Genes affected by deleterious GVs in three studies are given in bold print; each of these genes was mutated in at least one family of our study

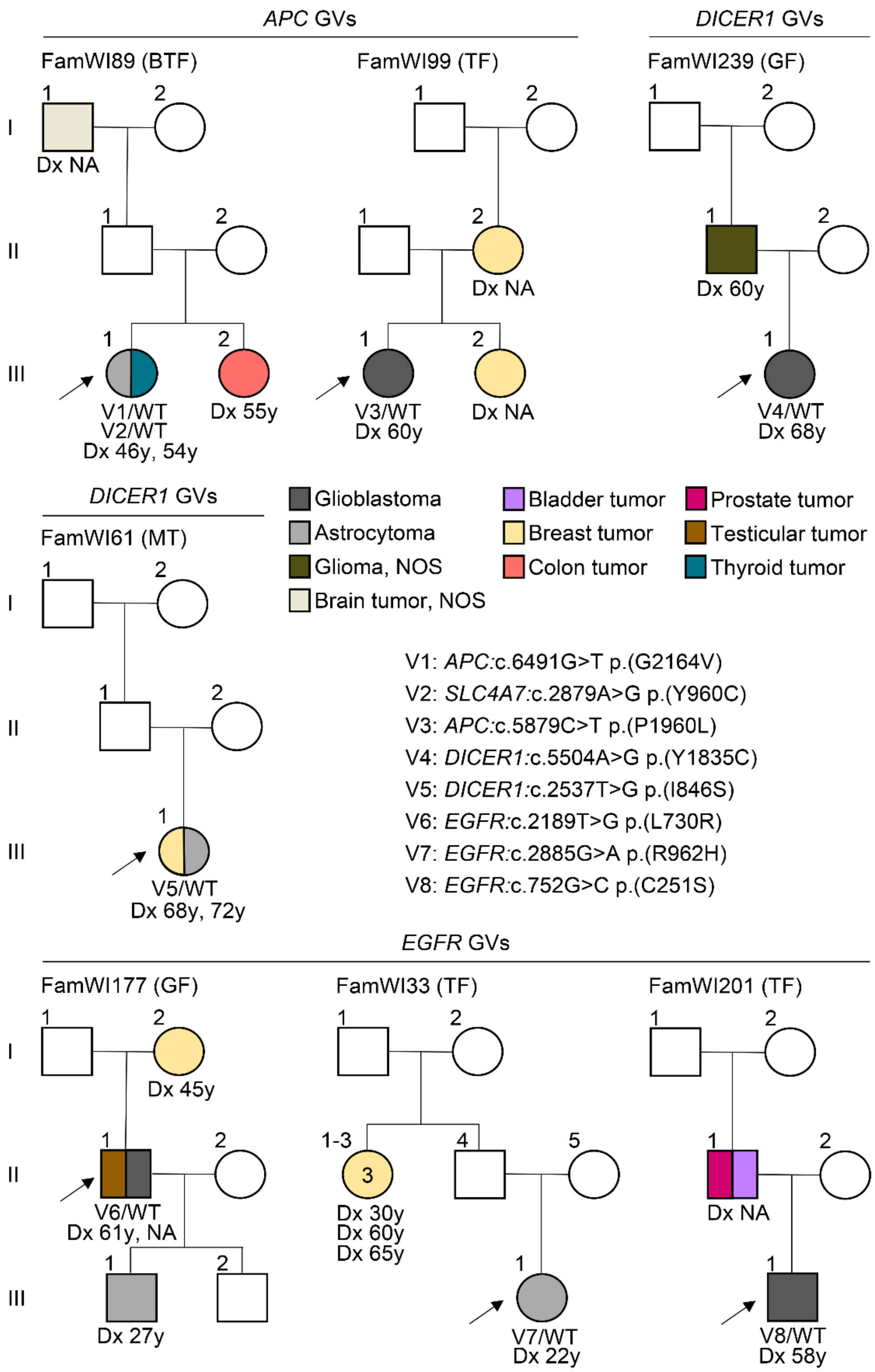

Supplementary Fig. 1 Pedigrees indicating familial and/or personal tumor spectrum of glioma patients with pathogenic GVs in the recurrently affected genes *APC* (and *SLC4A7* in one case), *DICER1*, or *EGFR*. Whether an individual is alive or deceased is not indicated. BTF, brain tumor family; Dx, age at diagnosis of primary tumor; GF, glioma family; GV, germline variant; MT, multiple tumors: glioma patient with ≥1 syn- or metachronous non-brain tumor but an unremarkable family history; NA, not available; NOS, not otherwise specified; TF, tumor family; V, variant; WT, wildtype; y, years

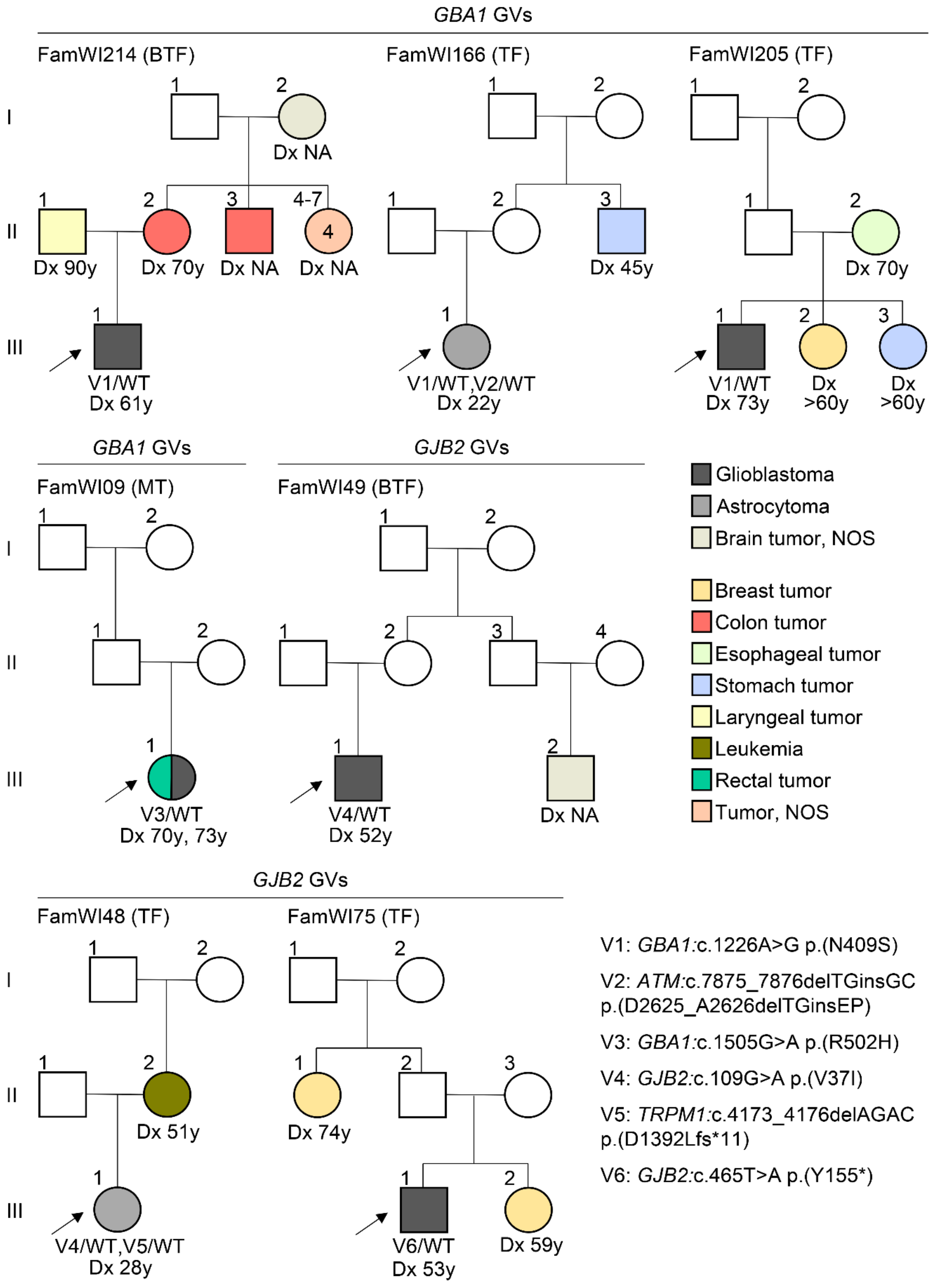

Supplementary Fig. 2 Pedigrees indicating familial and/or personal tumor spectrum of glioma patients with pathogenic GVs in the recurrently affected genes *GBA1* (and *ATM* in one case) or *GJB2* (and *TRPM1* in one case). Whether an individual is alive or deceased is not indicated. BTF, brain tumor family; Dx, age at diagnosis of primary tumor; GV, germline variant; MT, multiple tumors: glioma patient with ≥1 syn- or metachronous non-brain tumor but an unremarkable family history; NA, not available; NOS, not otherwise specified; TF, tumor family; V, variant; WT, wildtype; y, years

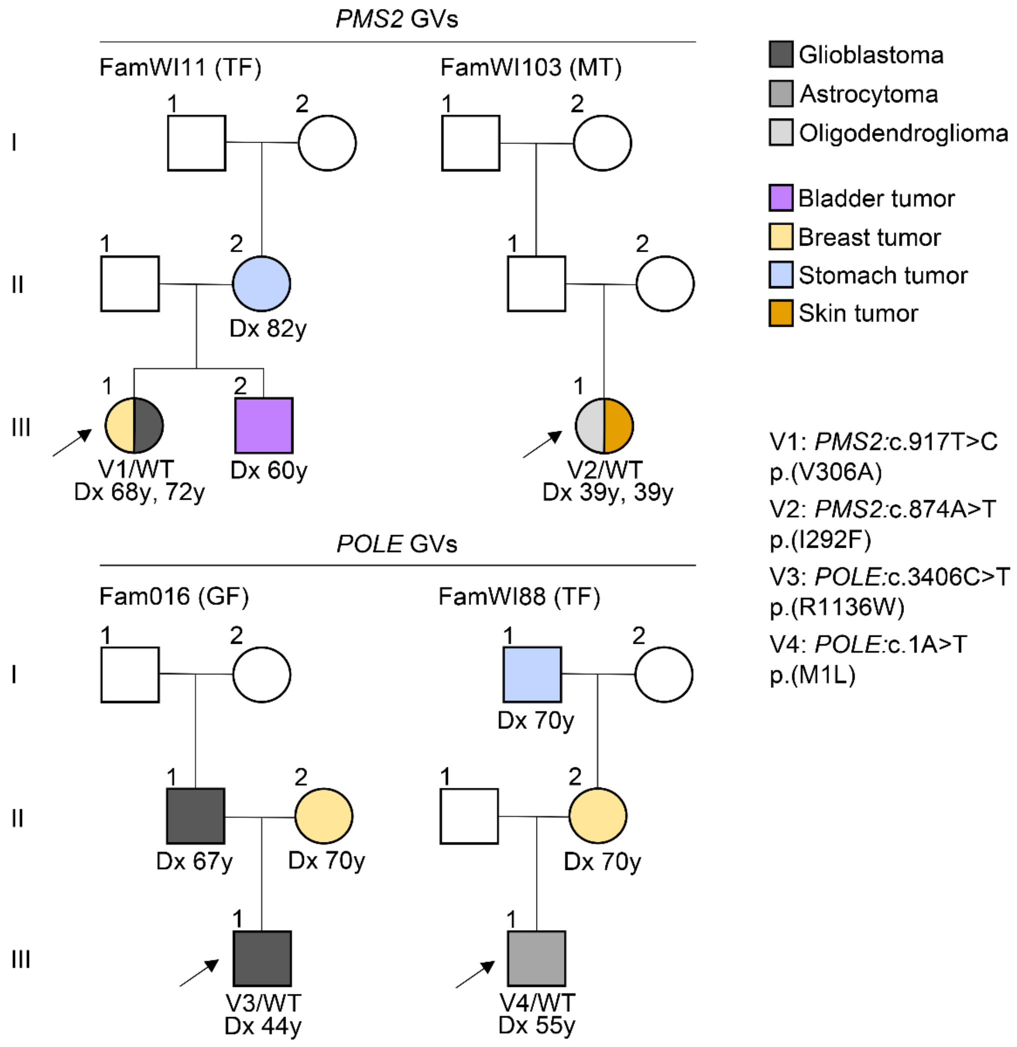

Supplementary Fig. 3 Pedigrees indicating familial and/or personal tumor spectrum of glioma patients with pathogenic GVs in the recurrently affected genes *PMS2* and *POLE*. Whether an individual is alive or deceased is not indicated. Dx, age at diagnosis of primary tumor; GF, glioma family; GV, germline variant; MT, multiple tumors: glioma patient with ≥1 syn- or metachronous non-brain tumor but an unremarkable family history; TF, tumor family; V, variant; WT, wildtype; y, years

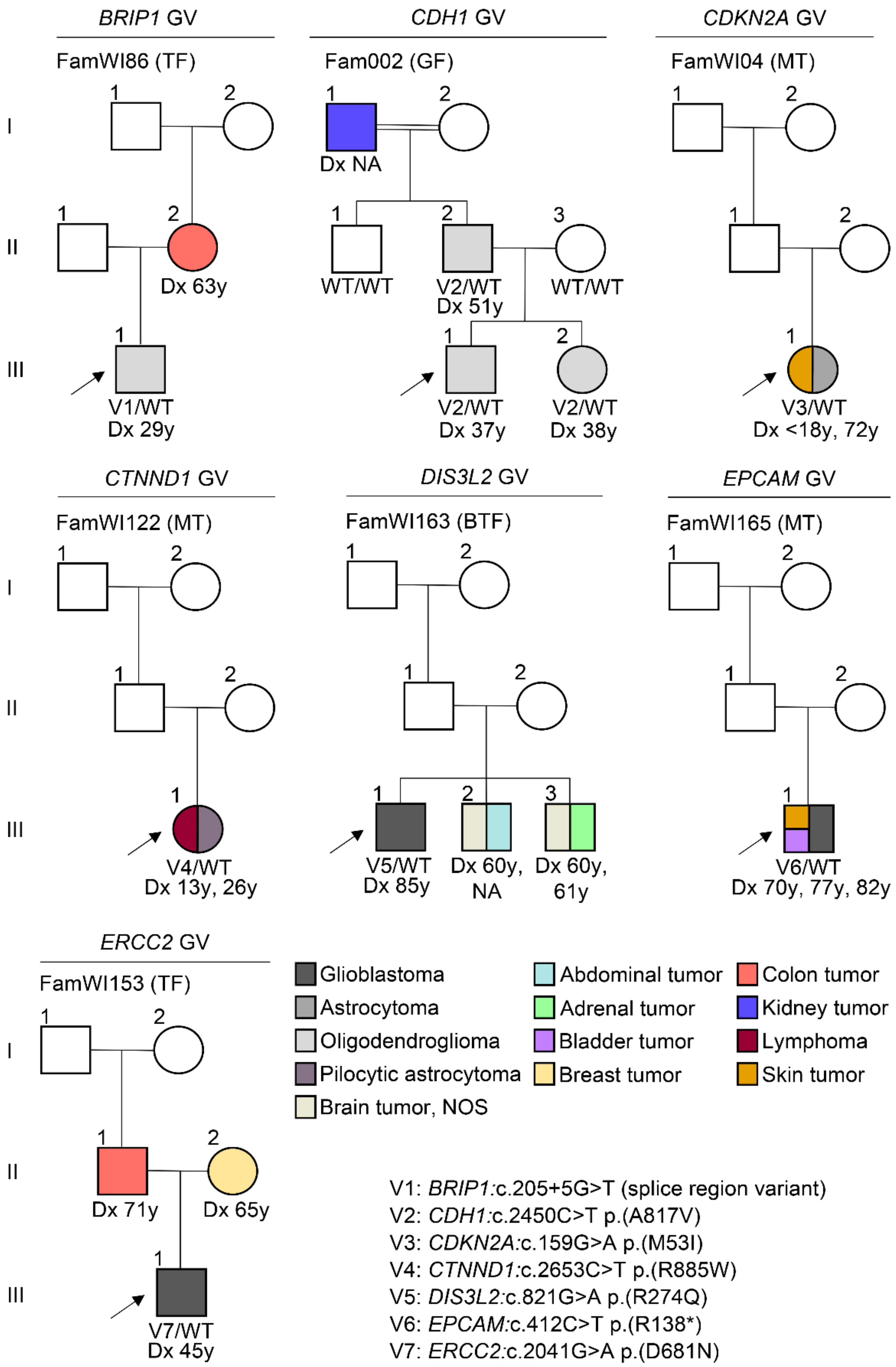

Supplementary Fig. 4 Pedigrees indicating familial and/or personal tumor spectrum of glioma patients with pathogenic GVs in *BRIP1*, *CDH1*, *CDKN2A*, *CTNND1*, *DIS3L2*, *EPCAM,* and *ERCC2*. A double horizontal line indicates consanguinity between two individuals. Whether an individual is alive or deceased is not indicated. BTF, brain tumor family; Dx, age at diagnosis of primary tumor; GV, germline variant; MT, multiple tumors: glioma patient with ≥1 syn- or metachronous non-brain tumor but an unremarkable family history; NA, not available; NOS, not otherwise specified; TF, tumor family; V, variant; WT, wildtype; y, years

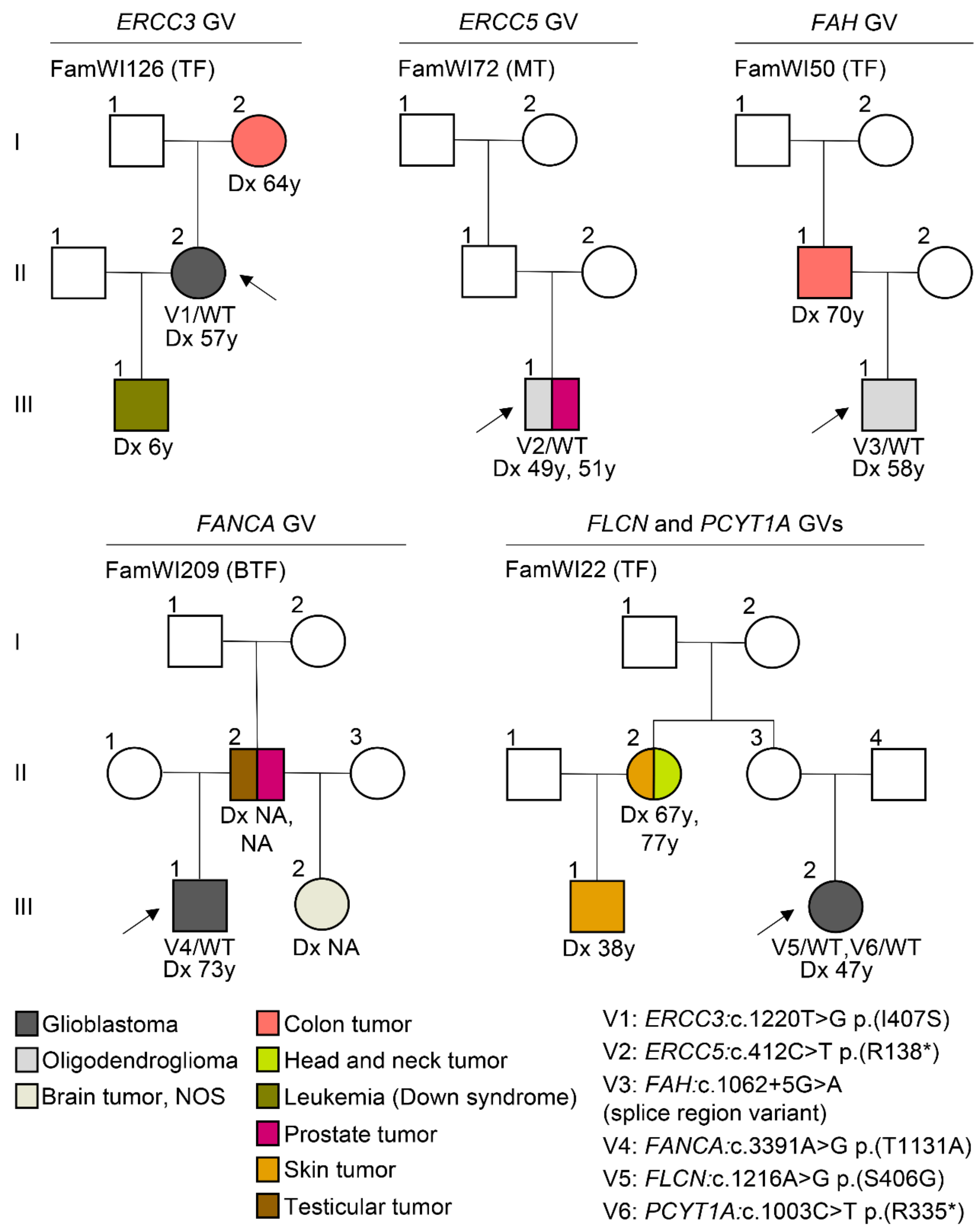

Supplementary Fig. 5 Pedigrees indicating familial and/or personal tumor spectrum of glioma patients with pathogenic GVs in *ERCC3*, *ERCC5*, *FAH*, *FANCA*, *FLCN*, and *PCYT1A*. Whether an individual is alive or deceased is not indicated. BTF, brain tumor family; Dx, age at diagnosis of primary tumor; GV, germline variant; MT, multiple tumors: glioma patient with ≥1 syn- or metachronous non-brain tumor but an unremarkable family history; NA, not available; NOS, not otherwise specified; TF, tumor family; V, variant; WT, wildtype; y, years

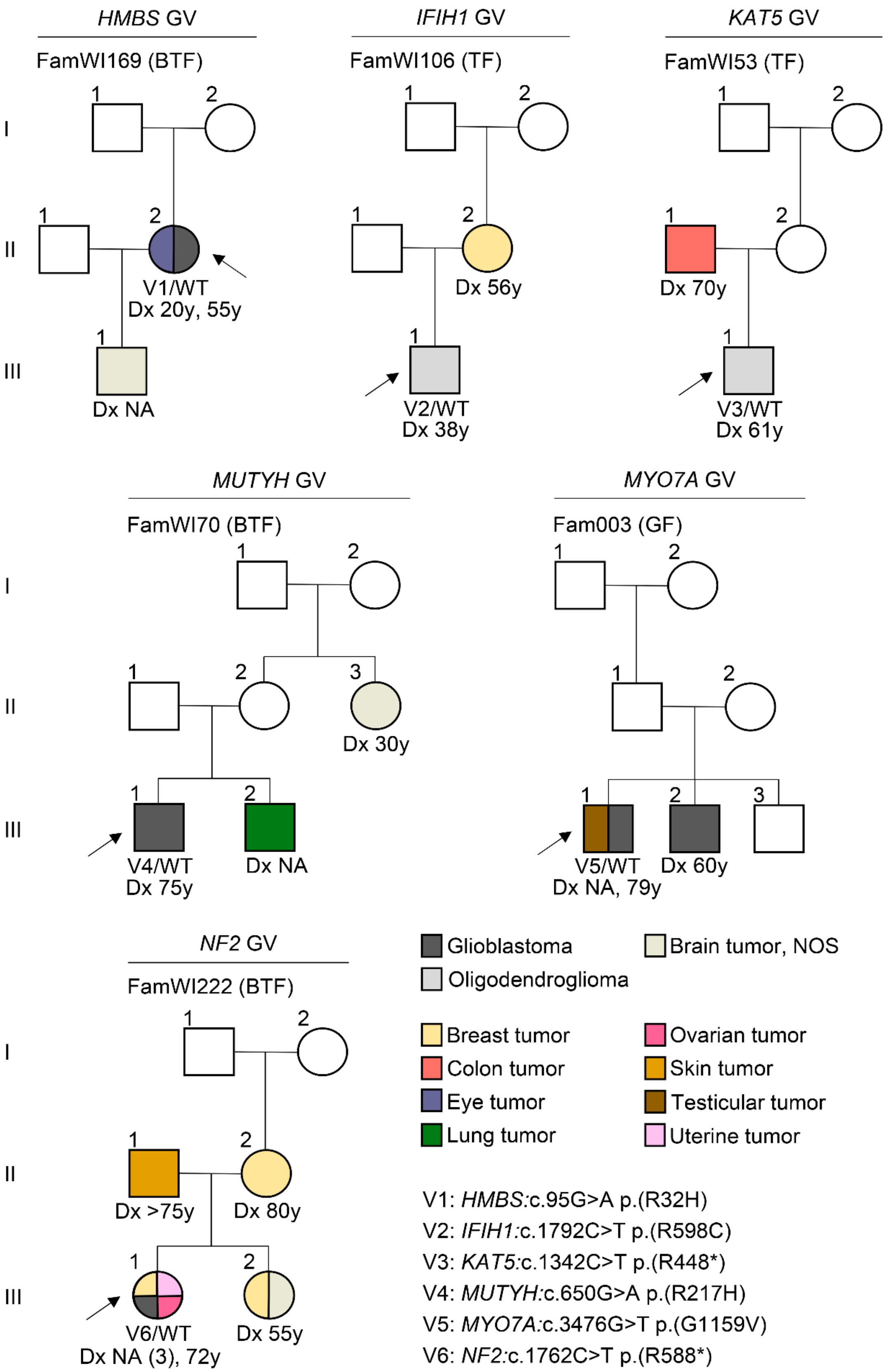

Supplementary Fig. 6 Pedigrees indicating familial and personal tumor spectrum of glioma patients with pathogenic GVs in *HMBS*, *IFIH1*, *KAT5*, *MUTYH*, *MYO7A*, and *NF2*. Whether an individual is alive or deceased is not indicated. BTF, brain tumor family; Dx, age at diagnosis of primary tumor; GF, glioma family; GV, germline variant; NA, not available; NOS, not otherwise specified; TF, tumor family; V, variant; WT, wildtype; y, years

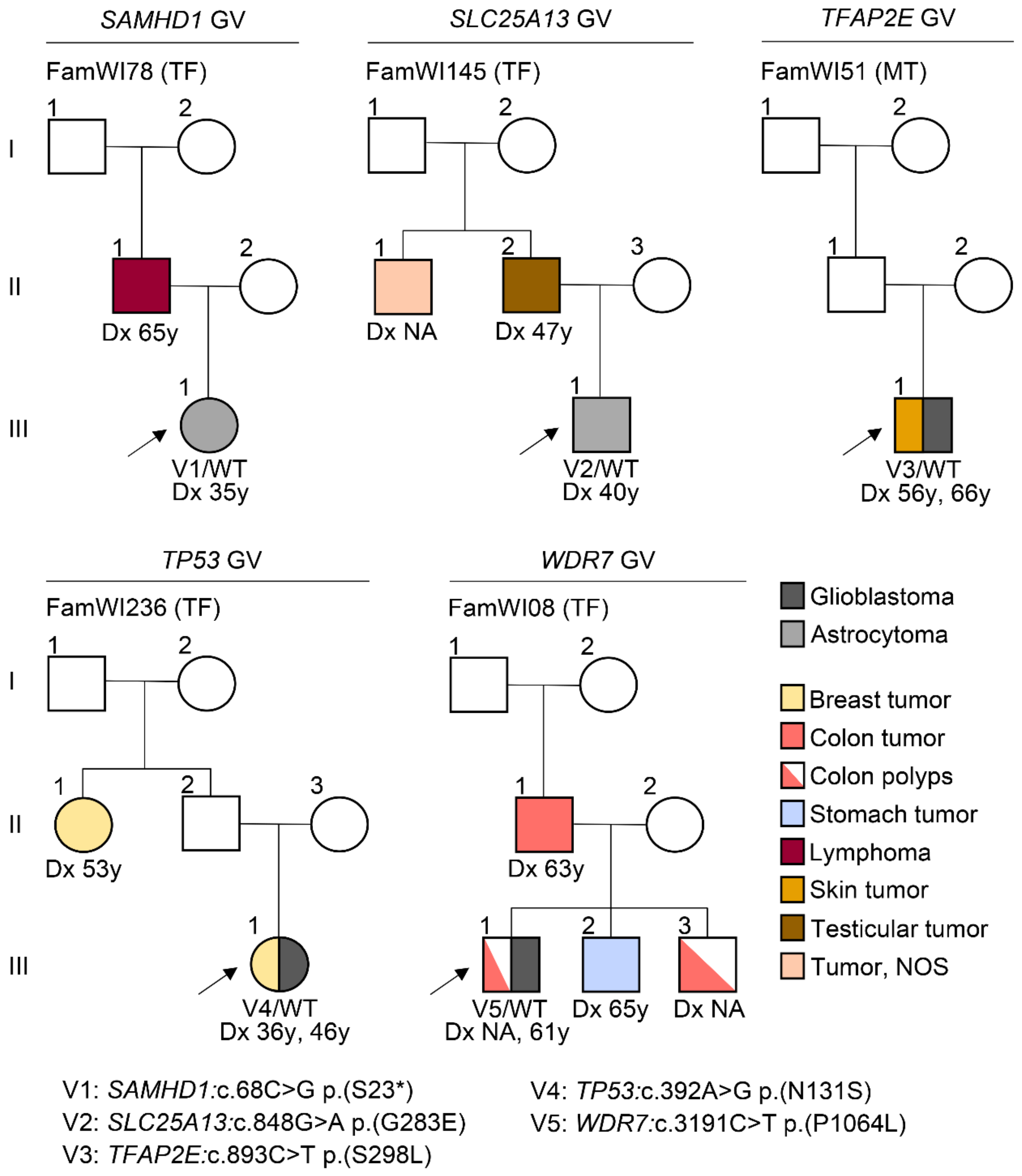

Supplementary Fig. 7 Pedigrees indicating familial and/or personal tumor spectrum of glioma patients with pathogenic GVs in *SAMHD1*, *SLC25A13*, *TFAP2E*, *TP53*, and *WDR7*. Whether an individual is alive or deceased is not indicated. Dx, age at diagnosis of primary tumor; GV, germline variant; MT, multiple tumors: glioma patient with ≥1 syn- or metachronous non-brain tumor but an unremarkable family history; NA, not available; NOS, not otherwise specified; TF, tumor family; V, variant; WT, wildtype; y, years

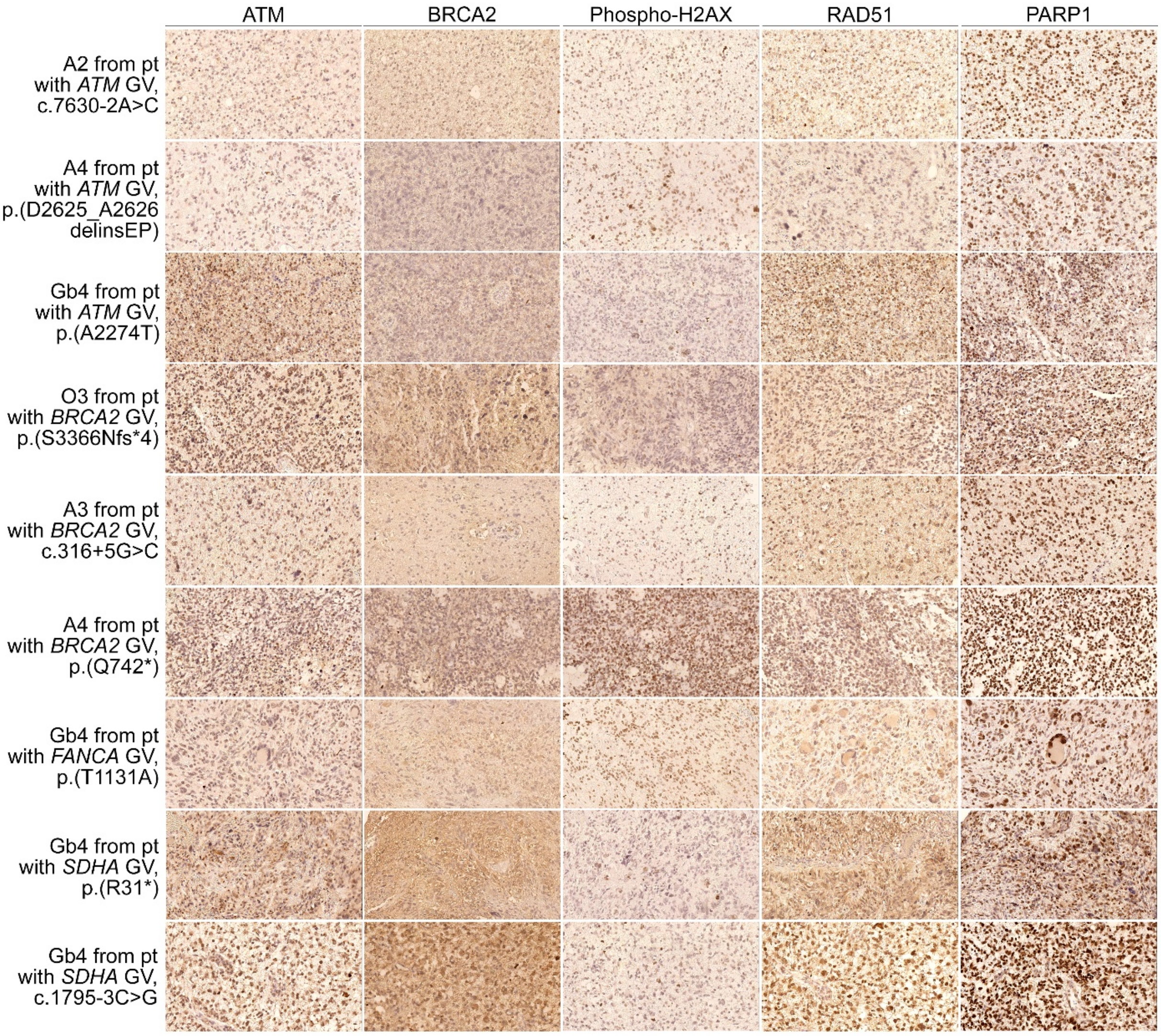

Supplementary Fig. 8 Expression analysis of ATM, BRCA2, phospho-H2AX, RAD51 and PARP1 on formalin-fixed paraffin-embedded glioma sections from nine patients with GVs in the CPGs *ATM*, *BRCA2*, *FANCA* and *SDHA* associated with DNA damage response by immunohistochemistry. For semi-quantitative determination of the immunoreactivity score of nuclear ATM, BRCA2, phospho-H2AX, RAD51 and PARP1 staining five fields per tumor were analyzed, one of which per stain and tumor is shown here. A2, astrocytoma, IDH-mutant, CNS WHO grade 2; A3, astrocytoma, IDH-mutant, CNS WHO grade 3; A4, astrocytoma, IDH-mutant, CNS WHO grade 4; CPG, cancer predisposition gene; O3, oligodendroglioma, IDH-mutant and 1p/19q-codeleted, CNS WHO grade 3; Gb4, glioblastoma, IDH-wildtype, CNS WHO grade 4; GV, germline variant; pt, patient

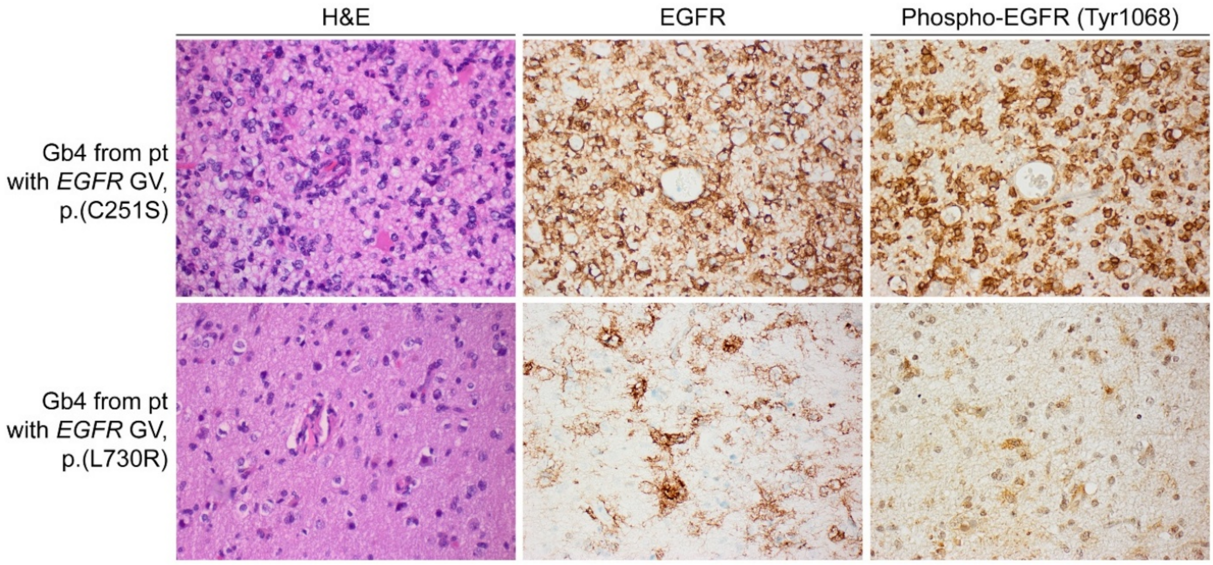

Supplementary Fig. 9 Expression analysis of EGFR and phospho-EGFR (Tyr1068) on formalin-fixed paraffin-embedded tumor sections from the two glioblastoma patients with GVs in *EGFR* by immunohistochemistry. EGFR is expressed and Tyr1068 phosphorylated in both glioblastomas. Gb4, glioblastoma, IDH-wildtype, CNS WHO grade 4; GV, germline variant; pt, patient

References

Bainbridge MN, Armstrong GN, Gramatges MM, Bertuch AA, Jhangiani SN, Doddapaneni H, Lewis L, Tombrello J, Tsavachidis S, Liu Y, Jalali A, Plon SE, Lau CC, Parsons DW, Claus EB, Barnholtz-Sloan J, Il'yasova D, Schildkraut J, Ali-Osman F, Sadetzki S, Johansen C, Houlston RS, Jenkins RB, Lachance D, Olson SH, Bernstein JL, Merrell RT, Wrensch MR, Walsh KM, Davis FG, Lai R, Shete S, Aldape K, Amos CI, Thompson PA, Muzny DM, Gibbs RA, Melin BS, Bondy ML; Gliogene Consortium. Germline mutations in shelterin complex genes are associated with familial glioma. *J Natl Cancer Inst*. 2015;107(1):384.

Beyer U, Brand F, Martens H, Weder J, Christians A, Elyan N, Hentschel B, Westphal M, Schackert G, Pietsch T, Hong B, Krauss JK, Samii A, Raab P, Das A, Dumitru CA, Sandalcioglu IE, Hakenberg OW, Erbersdobler A, Lehmann U, Reifenberger G, Weller M, Reijns MAM, Preller M, Wiese B, Hartmann C, Weber RG. Rare ADAR and RNASEH2B variants and a type I interferon signature in glioma and prostate carcinoma risk and tumorigenesis. *Acta Neuropathol*. 2017;134(6):905-922.

Brand F, Förster A, Christians A, Bucher M, Thomé CM, Raab MS, Westphal M, Pietsch T, von Deimling A, Reifenberger G, Claus P, Hentschel B, Weller M, Weber RG. FOCAD loss impacts microtubule assembly, G2/M progression and patient survival in astrocytic gliomas. *Acta Neuropathol*. 2020;139(1):175-192.

Brockschmidt A, Trost D, Peterziel H, Zimmermann K, Ehrler M, Grassmann H, Pfenning PN, Waha A, Wohlleber D, Brockschmidt FF, Jugold M, Hoischen A, Kalla C, Waha A, Seifert G, Knolle PA, Latz E, Hans VH, Wick W, Pfeifer A, Angel P, Weber RG. KIAA1797/FOCAD encodes a novel focal adhesion protein with tumour suppressor function in gliomas. *Brain*. 2012;135(Pt 4):1027-1041.

Catalano C, Paramasivam N, Blocka J, Giangiobbe S, Huhn S, Schlesner M, Weinhold N, Sijmons R, de Jong M, Langer C, Preuss KD, Nilsson B, Durie B, Goldschmidt H, Bandapalli OR, Hemminki K, Försti A. Characterization of rare germline variants in familial multiple myeloma. *Blood Cancer J*. 2021;11(2):33.

Choi DJ, Armstrong G, Lozzi B, Vijayaraghavan P, Plon SE, Wong TC, Boerwinkle E, Muzny DM, Chen HC, Gibbs RA, Ostrom QT, Melin B, Deneen B, Bondy ML; Gliogene Consortium; Genomics England Research Consortium; Bainbridge MN, Amos CI, Barnholtz-Sloan JS, Bernstein JL, Claus EB, Houlston RS, Il'yasova D, Jenkins RB, Johansen C, Lachance D, Lai R, Melin BS, Merrell RT, Olson SH, Sadetzki S, Schildkraut J, Shete S, Ambrose JC, Arumugam P, Bevers R, Bleda M, Boardman-Pretty F, Boustred CR, Brittain H, Brown MA, Caulfield MJ, Chan GC, Giess A, Griffin JN, Hamblin A, Henderson S, Hubbard TJP, Jackson R, Jones LJ, Kasperaviciute D, Kayikci M, Kousathanas A, Lahnstein L, Lakey A, Leigh SEA, Leong IUS, Lopez FJ, Maleady-Crowe F, McEntagart M, Minneci F, Mitchell J, Moutsianas L, Mueller M, Murugaesu N, Need AC,

O'Donovan P, Odhams CA, Patch C, Perez-Gil D, Pereira MB, Pullinger J, Rahim T, Rendon A, Rogers T, Savage K, Sawant K, Scott RH, Siddiq A, Sieghart A, Smith SC, Sosinsky A, Stuckey A, Tanguy M, Taylor Tavares AL, Thomas ERA, Thompson SR, Tucci A, Welland MJ, Williams E, Witkowska K, Wood SM, Zarowiecki M. The genomic landscape of familial glioma. *Sci Adv*. 2023;9(17):eade2675.

Crow YJ, Stetson DB. The type I interferonopathies: 10 years on. *Nat Rev Immunol*. 2022;22(8):471-483.

Förster A, Brand F, Banan R, Hüneburg R, Weber CAM, Ewert W, Kronenberg J, Previti C, Elyan N, Beyer U, Martens H, Hong B, Bräsen JH, Erbersdobler A, Krauss JK, Stangel M, Samii A, Wolf S, Preller M, Aretz S, Wiese B, Hartmann C, Weber RG. Rare germline variants in the E-cadherin gene CDH1 are associated with the risk of brain tumors of neuroepithelial and epithelial origin*. Acta Neuropathol.* 2021;142(1):191-210.

Jonsson P, Lin AL, Young RJ, DiStefano NM, Hyman DM, Li BT, Berger MF, Zehir A, Ladanyi M, Solit DB, Arnold AG, Stadler ZK, Mandelker D, Goldberg ME, Chmielecki J, Pourmaleki M, Ogilvie SQ, Chavan SS, McKeown AT, Manne M, Hyde A, Beal K, Yang TJ, Nolan CP, Pentsova E, Omuro A, Gavrilovic IT, Kaley TJ, Diamond EL, Stone JB, Grommes C, Boire A, Daras M, Piotrowski AF, Miller AM, Gutin PH, Chan TA, Tabar VS, Brennan CW, Rosenblum M, DeAngelis LM, Mellinghoff IK, Taylor BS. Genomic Correlates of Disease Progression and Treatment Response in Prospectively Characterized Gliomas. *Clin Cancer Res*. 2019;25(18):5537-5547.

McCormick EM, Keller K, Taylor JP, Coffey AJ, Shen L, Krotoski D, Harding B; NICHD ClinGen U24 Mitochondrial Disease Gene Curation Expert Panel; Gai X, Falk MJ, Zolkipli-Cunningham Z, Rahman S. Expert Panel Curation of 113 Primary Mitochondrial Disease Genes for the Leigh Syndrome Spectrum. *Ann Neurol*. 2023;94(4):696-712.

McDonald MF, Prather LL, Helfer CR, Ludmir EB, Echeverria AE, Yust-Katz S, Patel AJ, Deneen B, Rao G, Jalali A, Dhar SU, Amos CI, Mandel JJ. Prevalence of pathogenic germline variants in adult-type diffuse glioma. *Neurooncol Pract*. 2023;10(5):482-490.

Mitchell J, Camacho N, Shea P, Stopsack KH, Joseph V, Burren O, Dhindsa R, Nag A, Berchuck JE, O'Neill A, Abbasi A, Zoghbi AW, Alegre-Díaz J, Kuri-Morales P, Berumen J, Tapia-Conyer R, Emberson J, Torres JM, Collins R, Wang Q, Goldstein D, Matakidou A, Haefliger C, Anderson-Dring L, March R, Jobanputra V, Dougherty B, Carss K, Petrovski S, Kantoff PW, Offit K, Mucci LA, Pomerantz M, Fabre MA. Assessing the contribution of rare protein-coding germline variants to prostate cancer risk and severity in 37,184 cases. *Nat Commun*. 2025;16(1):1779.

Rahman N. Realizing the promise of cancer predisposition genes. *Nature*. 2014;505(7483):302-308.

Weber CAM, Krönke N, Volk V, Auber B, Förster A, Trost D, Geffers R, Esmaeilzadeh M, Lalk M, Nabavi A, Samii A, Krauss JK, Feuerhake F, Hartmann C, Wiese B, Brand F, Weber RG. Rare germline variants in POLE and POLD1 encoding the catalytic subunits of DNA polymerases ε and δ in glioma families. *Acta Neuropathol Commun*. 2023;11(1):184.
